# Molecular insights into the antimicrobial and cardiometabolic functions of *Lactobacillus crispatus* isolated from the reproductive tract microbiota of Indian women

**DOI:** 10.1186/s12929-025-01207-w

**Published:** 2026-01-05

**Authors:** Shriram Mahajan, N. Lekshmi, Proxima Dhiman, Manjari Gupta, Pallavi Mudgal, Rajni Yadav, Sudheer Arava, Shinjini Bhatnagar, Nitya Wadhwa, Yashwant Kumar, Daizee Talukdar, Bhabatosh Das, Sanjay K. Banerjee

**Affiliations:** 1 Department of Biotechnology, National Institute of Pharmaceutical Education and Research, Guwahati, Assam 781101 India; 2 BRIC-Translational Health Science and Technology Institute, Faridabad, Haryana India; 3 All India Institute of Medical Sciences, New Delhi, India

**Keywords:** Antimicrobial peptide, Anti-steatosis, Biotherapeutic, Cardioprotective, *Lactobacillus crispatus*, Probiotic

## Abstract

**Background:**

*Lactobacillus crispatus* is a dominant member of the healthy female reproductive tract microbiota, contributing to mucosal homeostasis and pathogen exclusion. Numerous studies have highlighted the protective effects of *L. crispatus* against both intestinal and genital infections. In the present study, we build on this foundation to investigate the broader health-promoting properties of *L. crispatus*, focusing on its antimicrobial and metabolic functions; and its protective roles in hepatic and cardiometabolic disorders.

**Methods:**

Three *L. crispatus* strains were selected from a panel of sixty isolates based on comprehensive genome mining analyses described in our previous publication. In the present study, we generated complete genome data for these three strains, and delineated biosynthetic pathways including their capacity for antimicrobial peptide production, lactic acid biosynthesis, short chain fatty acid synthesis and biogenic amine production. The antimicrobial activity of these isolates was assessed via agar well-diffusion assay and time-kill assay. Their ability to survive gastric pH and bile stress was evaluated through acid and bile salt tolerance assays. Further, to assess metabolic benefits, anti-steatotic and cardioprotective effects were examined in a preclinical diet-induced mouse model of cardiometabolic disorder.

**Results:**

Complete genome analysis of *L. crispatus* strains revealed multiple antimicrobial peptide (AMP) biosynthetic gene clusters, including several novel loci associated with bacteriocins. Metabolic profiling identified pathways for bile salt metabolism, folate biosynthesis and short chain fatty acids production. Cell-free culture supernatants exhibited broad-spectrum antibacterial activity, particularly against *Escherichia coli*, *Enterobacter hormaechei, Staphylococcus aureus* and *Staphylococcus haemolyticus*. Further, the strains tolerated gastric pH 2 and physiological bile stress of 0.3% suggesting potential for oral administration. In vivo, oral administration of *L. crispatus* (10^8^ CFU) daily for 2 weeks followed by twice-weekly for 12 weeks significantly reduced hepatic steatosis, improved insulin sensitivity and cardiac function in a diet-induced cardiometabolic disorder mouse model. This is the first report demonstrating the cardiometabolic protective potential of *L. crispatus*.

**Conclusions:**

*L. crispatus* confers diverse health benefits through pathogen resistance functions and modulation of host metabolic pathways. These findings support its potential as a novel biotherapeutic for preventing and managing hepatic and cardiometabolic disorders, extending its therapeutic relevance beyond reproductive health.

**Supplementary Information:**

The online version contains supplementary material available at 10.1186/s12929-025-01207-w.

## Introduction

The human microbiome plays a central role in maintaining host health, with commensal microorganisms contributing to immune regulation, barrier integrity, and resistance to pathogenic colonization. In the female reproductive tract, *Lactobacillus crispatus* is one of the most dominant and beneficial species, commonly associated with vaginal eubiosis and reduced risk of infection-related complications such as bacterial vaginosis, sexually transmitted infections, and preterm birth [1, 2]. Its dominance is considered a marker of a stable and protective microbial environment, largely attributed to its capacity to produce lactic acid and other antimicrobial compounds, thereby contributing to mucosal homeostasis and pathogen exclusion [3, 4]. Several strains of *L. crispatus* have been rigorously studied and demonstrate strong promise in supporting vaginal and reproductive health [5, 6].

Despite extensive evidence supporting the importance of *L. crispatus* in reproductive health, its broader physiological benefits, particularly its potential impact on systemic metabolic processes and chronic disease risk i.e., cardiometabolic health remain underexplored. Metabolic dysfunction associated steatotic liver disease (MASLD), is an increasingly prevalent hepatic condition characterized by excessive fat accumulation in the liver. MASLD is strongly linked to cardiometabolic syndromes which include obesity, insulin resistance, dyslipidemia, type 2 diabetes mellitus and cardiac dysfunction [7, 8]. Despite its growing global burden, effective therapeutic strategies remain limited, with current management largely reliant on lifestyle interventions. Recent studies have underscored the gut-liver axis and highlighted intricate links between the microbiome and host metabolic pathways, including those influencing insulin sensitivity, hepatic lipid accumulation and inflammation, and cardiovascular function [9–11]. These findings have propelled interest in probiotic-based interventions aimed at restoring microbial balance and modulate host metabolism. However, many commercially available probiotics aim at only maintaining general health and consist of poorly characterized strains with limited or inconsistent efficacy, reducing their translational relevance. Repurposing well characterized human-derived *Lactobacillus* species offers a promising approach to developing biotherapeutics with safety and host compatibility. In our previous study, we had identified sixty *L. crispatus* isolates from 133 *Lactobacillus* isolates cultured from high vaginal swab samples of healthy pregnant Indian women enrolled in the GARBH-Ini cohort [12]. Draft genome sequencing and comparative genomic analysis revealed that the vaginal *L. crispatus* isolates harbored multiple genes associated with pathogen resistance, adhesion, acid tolerance and immunomodulation indicating probiotic potential [12]. In this context, we wanted to explore the systemic effects of these vaginally isolated *L. crispatus* strains beyond its native niche.

In the present study, three *L. crispatus* isolates exhibiting superior pathogen resistance as identified by our previous study were selected for detailed characterization of their antimicrobial and metabolic capacities by complete genome sequencing and functional studies. Further, we have evaluated the protective role of the vaginally isolated *L. crispatus* strain in a preclinical model of diet-induced metabolic dysfunction. Collectively, this work aims to elucidate the potential of translating *L. crispatus* as a novel biotherapeutic candidate with dual benefits in promoting both mucosal and systemic health.

## Materials and methods

### Bacterial strains and culture conditions

The *L. crispatus* strains S10-5-C2-2, S9-4-C11 and S7-7-C9, isolated from the vaginal microbiota of healthy Indian women enrolled in the GARBH-Ini cohort, were selected for detailed investigation based on the results of our previous study [12]. The probiotic strain *Lactiplantibacillus rhamnosus* ATCC 53103, which has been widely used in commercial probiotic products was used as positive control to evaluate the gastric pH and bile salt tolerance of the *L. crispatus* strains. The *Lactobacillus* strains were routinely cultivated on de Man, Rogosa, and Sharpe (MRS) agar (Sigma-Aldrich, Cat no.69964) and in MRS broth (Sigma-Aldrich, Cat no.69966) supplemented with 0.001% Tween 80 (Sigma-Aldrich, Cat no. P8074) incubated at 37 °C under anaerobic conditions (85% N_2_ + 10% CO_2_ + 5% H_2_) for 24–48 h. For long term storage, bacterial cultures were preserved at −80 °C in MRS medium containing 15% sterile glycerol or in lyophilized form with 10% w/v sucrose as the lyophilization matrix. The Gram- negative and Gram-positive bacterial pathogens used in the study were routinely cultured on Luria Bertani (LB) agar (Sigma-Aldrich, Cat no. L2025) and LB broth (Sigma-Aldrich, Cat no. L1900), respectively. The fungal isolates used in the study were cultured on Potato Dextrose agar (PDA) (HiMedia, Cat no. MH096). The antimicrobial activity of the *L. crispatus* strains were assessed by the well diffusion assay on Mueller Hinton agar (MHA) (Sigma-Aldrich, Cat no. 70191). The list of bacterial and fungal isolates used in the study and their culture conditions are detailed in supplementary Table 1.

### DNA extraction, whole genome sequencing, assembly and annotation

Genomic DNA from the selected strains was extracted using the GenElute^™^ Bacterial Genomic DNA Isolation Kit (Sigma-Aldrich, Cat. No. NA2120). Whole genome sequencing was carried out on the Oxford Nanopore platform (Oxford, UK). High-quality short reads previously generated via Illumina sequencing [12] and long reads obtained from Oxford Nanopore in this study were used for hybrid genome assembly with Unicycler v0.4.8, which integrates both read types to produce high-contiguity assemblies [13]. Genome quality and contamination levels were assessed using CheckM program version v1.1.3, applying a contamination cutoff of < 5% and completeness cutoff > 95% [14]. Annotations of the hybrid genome were performed using the Rapid annotation using Subsystem technology (RAST) [15].

### In silico probiotic characterization: safety and functionality

In silico probiotic characterization of the isolates were conducted using the ProbioMinServer [16]. The ProbioMinServer integrates robust bioinformatic pipelines such as Mash v2.3 to confirm the bacterial isolate, and EggNOG-mapper v2 for genome annotation with > 90% identity and > 80% coverage as parameters [17]. To assess antimicrobial resistance (AMR) the Comprehensive Antibiotic Resistance Database (CARD) Variants v4.0.0 [18] and ResFinder v4.0 [19] was used. Further, pathogenic potential is assessed using VirulenceFinder v2.0.3 [20]. Additionally, safety profiles of the isolates were also evaluated by screening for mobile genetic elements using PlasmidFinder v2.0.1 [21] and Phigaro v2.3.0 [22]. Combining the results of each assessment, the platform computes the probiotic potential risk score (PPRS) which categorizes the isolate’s safety. A score of ≥ 6 denotes high risk while a score between 4 and 6 denotes medium risk and ≤ 4 denotes low risk.

### Metabolic function profiling and biosynthetic gene cluster (BGCs) identification

Metabolic reconstruction from genomic data was performed using KEGG and COG [23] databases. Key pathways related to biogenic amine production, bile salt metabolism, vitamin biosynthesis, short chain fatty acid production and folate biosynthesis were examined. The complete genomes of the *L. crispatus* strains S10-5-C2-2, S9-4-C11 and S7-7-C9 were mined to detect potential primary or secondary metabolite biosynthesis gene clusters (BGCs) using antiSMASH v7.0.0 [24] and gutSMASH v1.0.0 [25].

### Functional evaluation of pathogen resistance function

The antibacterial activity of the cell free supernatant (CFS) of the three *L. crispatus* strains S10-5-C2-2, S9-4-C11 and S7-7-C9 were evaluated as previously described [12] against a panel of different Gram- negative and Gram- positive pathogens including clinically relevant ESKAPE pathogens. In addition to pathogenic bacterial isolates, the antibacterial effects of the CFS were also tested against a range of fungal pathogens and other *Lactobacillus* spp (Supplementary table S1). Briefly, the CFS were collected by incubating the *L. crispatus* strains for 48 h followed by centrifugation at 10,000 rpm for 10 min at 4 °C. The pH of the resulting supernatant was measured and then sterilized using a 0.22 µm filter prior to performing the well diffusion assay. Bacterial pathogens were cultured in LB broth and fungal pathogens in Potato Dextrose broth until the mid-log phase and swabbed onto the MHA plate and PDA plate respectively. Likewise, the commensals, *Lactobacillus* spp. were cultured in MRS broth and swabbed on to MRS agar. Wells of 8 mm diameter were punched into the agar, and 100 µl the filter sterilized CFS were added to each well. The plates were incubated at 37 °C and zones of inhibitions were measured at designated time intervals. As controls, MRS broth adjusted to the same pH as the CFS using lactic acid was used as a pH control to normalize the pH dependent antimicrobial activity, while untreated MRS broth served as the negative control. The experiment was conducted in triplicates to ensure reproducibility.

To assess the direct antibacterial activity of *L. crispatus* CFS against selected Gram-negative and Gram-positive bacterial pathogens, a time-kill assay was performed with modifications to previously described protocols [26]. Pathogens were inoculated at an initial inoculum of 10^7^–10^8^ incubated in MRS broth served as the negative control while MRS broth pH adjusted with lactic acid was used as the positive control. All suspensions were incubated at 37 ℃ with agitation at 180 rpm. At designated time points, T0, T1, T2 and T3, samples were collected, serially diluted and plated on Luria Bertani agar (LA) to assess pathogen viability. Bactericidal activity was determined when there is a ≥ 3 log₁₀ reduction in CFU/mL of the pathogen from the initial inoculum. The experiment was performed in triplicate for reproducibility.

### Gastric pH tolerance and bile salt tolerance assay

To evaluate the potential of these *L. crispatus* isolates for oral administration, their tolerance to surpass the gastric pH and bile salt stress were evaluated as previously described with some modifications [27]. The *L. crispatus* strains were grown to an optical density (OD) that corresponds to 1X10^9^ colony forming unit (CFU) in MRS broth. The cultures were then centrifuged at 6000 rpm for 10 min at 4 ℃ and the resulting pellet was resuspended in MRS broth that had been adjusted to a pH 2 and 3 using 1N hydrochloric acid (HCl). Pellets resuspended in MRS broth without pH adjustment served as the positive control. The samples were incubated anaerobically at 37 ℃ without agitation and at intervals T0, T1, T2, T3, T16, T24 h, viable cell counts were determined by serial dilution and spotting on MRS agar. CFUs were enumerated after 48 h of incubation at 37 °C anaerobically and the survivability of the strains under gastric pH conditions was determined. Similarly, pellets were resuspended in MRS broth, supplemented with bile salt sodium deoxycholate at concentrations 0.15% and 0.3% and processed in the same manner. *L. rhamnosus* ATCC 53103 was used as the positive control. *L. rhamnosus* ATCC 53103 was used as the positive control as it is a well-characterized probiotic strain with robust gastric pH and bile salt tolerance, providing a gold-standard comparator to evaluate the probiotic functions of the test strains. Moreover, there is no well characterized *L. crispatus* reference strain that has been extensively used in gastric acidity and bile stress tolerance studies. The experiments were conducted in biological triplicates.

### Lyophilization and stability evaluation of *Lactobacillus crispatus*

To evaluate the long-term stability of *L. crispatus* in its lyophilized form, the selected strains S10-5-C2-2, S9-4-C11 and S7-7-C9 were subjected to freeze-drying using sucrose as the cryoprotectant. Briefly, *L. crispatus* strains were grown under anaerobic conditions in MRS broth at 37 °C. Cells were harvested by centrifugation at 6000 rpm for 20 min at 4 °C, washed twice with sterile phosphate-buffered saline (PBS), pH 7.4 and resuspended in a cryoprotective solution containing 10% (w/v) sterile-filtered sucrose. The cell suspensions were aliquoted into sterile falcons and lyophilized using a freeze dryer under standard conditions (− 80 °C, < 0.1 mbar). Lyophilized samples were stored at three different temperatures (room temperature, 4°C and − 20 °C) to assess the impact of storage conditions on bacterial viability. Viability was monitored for 3 months at regular intervals (Day 0, D7, D14, D28, up to D98). For each time point, rehydration of the samples was done with sterile anaerobic MRS broth, and viable cell counts were determined by serial dilution and spotting on MRS agar. CFUs were enumerated after 48 h of incubation at 37 °C anaerobically. The assay was conducted in triplicate for each condition to ensure reproducibility.

### Animals and experimental design

We received approval from the Institutional Animal Ethical Committee (IAEC), National Institute of Pharmaceutical Education and Research, Guwahati, to conduct the animal experiment (NIPER/BT/2022/68). C57BL6 male mice aged between 8 and 10 weeks (weight 20 ± 2 g) were obtained from Rodent Research India Pvt. Ltd. Mice were housed at NIPER, Guwahati in cages (six mice per group) under controlled environmental conditions such as temperature of 22–25 ℃ and humidity of 40–60% within a 12 h light/dark cycle. Animals had ad libitum access to standard pellet diet and purified drinking water through-out the study until otherwise described. Cardiometabolic disorder was induced in mice by chronic feeding of a choline-deficient high-fat (CDFD) diet obtained from Research Diet, USA. The weights of the mice were measured at the beginning and weekly thereafter. After 1 week of quarantine, the C57BL6 were divided into three different groups (N = 6 per group) as follows: (i) Normal chow diet with oral administration of saline for 2 weeks daily, followed by administration at 2-day intervals for an additional 12 weeks, (ii) CDHF diet with oral administration of saline for 2 weeks daily, followed by administration at 2-day intervals for an additional 12 weeks (iii) CDHF diet for 12 weeks with oral administration of *L. crispatus* strain S10-5-C2-2 (10^8^ CFU) for 2 weeks daily, followed by administration at 2-day intervals for an additional 12 weeks.

### Measurement of intraperitoneal glucose tolerance test (IPGTT)

The mice were fasted overnight for approximately 12–14 h by placing them in clean cages without access to food. The tip of each mouse’s tail was trimmed using a sterilized scalpel blade, and the initial drop of blood was discarded. A small blood sample (< 5 μl) was then applied to the glucose test strip for measurement with a glucometer, which served as the baseline glucose level (time = 0). Subsequently, the mice received an intraperitoneal injection of a 20% glucose solution at a dose of 2 gm of glucose per kilogram of body weight. Blood glucose concentrations were then determined at 15, 30, 60, and 120 min following the glucose administration using an Accu-Chek glucometer. Line graphs were plotted between blood glucose levels and time points to measure the area under the curve [28, 29].

### Histopathological assessment

Liver and heart tissues from mice (N = 3/group) were fixed in 10% phosphate-buffered formalin for 48 h. Formalin-fixed liver tissue was processed and embedded in paraffin. Paraffin sections (5 µm) were cut using a microtome and mounted on glass slides. Hematoxylin and Eosin (H & E) staining was performed to examine the tissue morphology and damage. Masson’s trichrome staining (MT) was performed to examine the fibrosis. Stained sections were viewed under a light microscope. H & E-stained sections and the extent of fibrosis from Masson’s trichrome-stained sections were quantified using ImageJ software. NAFLD activity score and fibrosis stage were evaluated by an expert pathologist according to the NASH CRN scoring system.

### Echogenicity in mice

Fat accumulation in mice liver was evaluated by imaging the liver using the Vevo LAZR-X 3100 system (Fujifilm VisualSonics Inc., Toronto, ON, Canada). Mice were first anesthetized and then positioned supine on a heated platform (maintained at 37 °C) to ensure thermal stability during imaging. The animals were gently secured on a three-axis micro-positioning stage to facilitate precise alignment of the ultrasound beam. A 20–46-MHz linear array transducer (MX400) was employed along with a high-conductivity ultrasound gel to acquire high-resolution two-dimensional (B-mode) images. Imaging was carried out using the pre-set "Mouse Abdominal (Small Animal)" protocol, specifically utilizing the liver preset view. All echocardiographic data were analyzed using VevoLab software (version 3.2.2).

### Echocardiography in mice

Mice cardiac imaging was performed using the Vevo LAZR-X 3100 system (Fujifilm VisualSonics Inc., Toronto, ON, Canada) to evaluate both structural and functional parameters of the mice heart. Mice were first anesthetized and then positioned supine on a heated platform (maintained at 37 °C) to ensure thermal stability during imaging. The animals were gently secured on a three-axis micro-positioning stage to facilitate precise alignment of the ultrasound beam. A 20–46-MHz linear array transducer (MX400) was employed along with a high-conductivity ultrasound gel to acquire high-resolution two-dimensional and M-mode images. Imaging was carried out using the pre-set "Mouse Cardiology (Small Animal)" protocol, specifically utilizing the parasternal long-axis (PSALX) view. All echocardiographic data were analyzed using VevoLab software (version 3.2.2).

### Metabolomics study

Metabolite extraction and data acquisition was done using previously established protocols described in Gautam et al. [30]. In brief, 100 μL of the serum samples and 400μL of chilled methanol were used for total metabolite extraction. After brief vortexing, sample were kept on ice for 30 min and followed by that centrifugation was done at 1000 rpm, 4 degree centigrade for ten minutes and supernatant were collected. These samples after drying were reconstituted in 50% water: methanol solution, and data acquisition was done using Orbitrap mass spectrometry with an ESI source and coupled with ultra-performance liquid chromatography. Metabolite separation was done using C18 (HSS T3) and HILIC (XBridge BEH Amide) columns on UPLC ultimate 3,000 system maintained at 40 °C, respectively. A gradient of mobile phase A consisting of 99.9% water and 0.1% formic acid and mobile phase B (99.9% methanol and 0.1% formic acid) was used as a mobile phase for the C18 column. For the separation in HILIC column, 20 mM ammonium acetate in water (pH 9.0) served as mobile phase A and 100% acetonitrile served as mobile phase B. For separation in the reverse phase, a gradient from 1 to 99% mobile phase B over 14 min (flow rate of 0.3 ml/min) was set and for the C18 column while 85% to 10% mobile phase B over 16 min (flow rate of 0.2 ml/min) was used for HILIC column. A 5 µl sample volume was injected into the column and for data acquisition. The Orbitrap Fusion Tribrid Mass Spectrometer (Thermo-Scientific) equipped with a heated electrospray ionization (HESI) source was used for the data acquisition using the following settings: 4000 positive mode spray voltage, 35,000 V for negative mode, 60–900 m/z mass range, AGC (Automatic gain control) was targeted at 100,000 ions. For data acquisition, 120,000 resolutions in MS1 mode and 30,000 resolutions in data-dependent MS2 scan mode were used. For MS, 50 ms was used as the maximum injection time while for MS/MS, an AGC target of 20,000 ions and a maximum injection time of 60 ms were used. The untargeted workflow of Tidymass software for metabolomics was used at default settings for acquiring and analyzing the data [31]. The mass, fragmented ions pattern, and retention time of identified compounds were compared with our in-house metabolite library and online spectral library search (in-house, MS-DIAL, HMDB, MASS Bank) to generate the list of identified metabolites. Further metabolomics data was analyzed with the help of MetaboAnalyst 6.0 through enrichment pathway analysis.

### Western blot analysis

Mice liver tissues were homogenized using RIPA lysis buffer with 1% sodium deoxycholate, 1 mM sodium orthovanadate, 50 mM sodium fluoride, 1 mM phenylmethanesulfonylfluoride and 1% protease inhibitor cocktail (Cell Signaling, Beverly, MA, USA). After homogenization, samples were sonicated and protein concentration was determined by the Bradford Protein Assay (Himedia, India). Protein samples were diluted in the loading buffer and denatured at 95 °C for 5 min. Proteins were run in SDS-PAGE and transferred onto PVDF membranes (Bio Rad, USA). TBS buffer containing 5% BSA and 0.1% Tween 20 (Sigma Aldrich, Darmstadt, Germany) was used to block the membrane. The membranes were then incubated with primary antibodies: AMPKa1/AMPKa2 Rabbit pAb (A17289); Phospho-AMPKa1/AMPKa2-T183/T172 Rabbit pAb (AP1171); PGC1α Rabbit pAb (A12348); PPARα Rabbit pAb (A18252); SIRT1 (D1D7) Rabbit mAb 9475; GAPDH Rabbit mAb (A19056) overnight at 4 °C and washed thrice for 5 min each before incubating with secondary antibody, HRP-conjugated anti-Rabbit (#AS014, ABclonal, USA) (1:10000). Chemiluminescence imaging was performed using the VILBER Imaging System (VILBER, USA).

### Statistical analysis

All statistical analyses were performed using GraphPad Prism version.10.9. (GraphPad Software Inc.) and experimental data of at least three independent experiments have been displayed with mean and standard deviation (SD). One way ANOVA was performed for analyzing the results. Statistical significance was accepted at p < 0.05. *p < 0.05, **p < 0.01, ***p < 0.001and ****p < 0.0001.

## Results

### Genomic insights into the safety and antimicrobial potential of the *Lactobacillus crispatus* strains reveal probiotic traits and pathogen resistance

We previously isolated, sequenced, and performed comparative genomic analyses of sixty *L. crispatus* strains obtained from high vaginal swab samples of healthy Indian women of reproductive age enrolled in Phase I of the GARBH-Ini cohort study [12]. Among the strains analyzed, *L. crispatus* S10-5-C2-2 (MGL39), S9-4-C11(MGL45), and S7-7-C9 (MGL46) emerged as promising candidates for further detailed investigation due to their multiple genomic signatures associated with pathogen resistance and ability to resist *Gardnerella vaginalis* ATCC 14018, *E. coli* ATCC 25922 and *K. pneumoniae* ATCC 1705 [12].

In the present study, complete genome sequencing of these three *L. crispatus* isolates was performed using a hybrid assembly approach that combined Illumina and Oxford Nanopore sequencing technologies to investigate further on its metabolic and biosynthetic functions. The complete genome sizes of the strains S10-5-C2-2, S9-4-C11 and S7-7-C9 were 2.24 Mb, 2.35 Mb and 2.33 Mb respectively (Supplementary Fig. 1A). The isolates also harbored one plasmid each of 19, 564 bp, 17, 388 bp and 17, 388 bp in S10-5-C2-2, S9-4-C11 and S7-7-C9 respectively which shared > 99% sequence identity with plasmid pLC2029 from *L. crispatus* 2029, isolated from the genital tract of healthy women. Important features of the three strains are summarized in Table 1. Functional classification of the *L. crispatus* ORFeome based on the eggNOG database predicted ORFs encoding proteins of different functions. The predicted ORFs were assigned to 10 functional groups. In particular, the highest number of predicted proteins identified in all three *L. crispatus* strains were identified to be involved in metabolism, protein processing and DNA processing followed by stress response and defense mechanisms (Supplementary Fig. 1B). Safety assessment via the ProbioMinServer yielded a PPRS score of ‘0’ for all the three strains. Resistome analysis using CARD and ResFinder identified no strict matches for AMR genes. Similarly, evaluation of virulence factors revealed no pathogenic traits in the strains, supporting their suitability as safe probiotic candidates.

**Table 1:** Genomic features of the *L. crispatus* strains evaluated in the study

Strain name	S10-5-C2-2	S9-4-C11	S7-7-C9
Contig No	2	2	2
Size (bp)	23,50,586	24,61,568	24,46,573
GC (%)	37.23	37.22	37.2
CDS	2358	2498	2486
rRNA	12	18	15
tRNA	69	72	69
tmRNA	1	1	1
Proteins with functional assignment	1,733	1,749	1,741
Hypothetical proteins	650	776	769
Plasmids	1 (19, 564 bp)	1 (17, 388 bp)	1 (17, 388 bp)
AMR genes	No hit	No hit	No hit
Virulence factors	No hit	No hit	No hit
Prophages	Siphoviridae, Myoviridae	Siphoviridae, Myoviridae	Siphoviridae, Myoviridae
Probiotic potential risk score	0	0	0
Bacteriocin	Gassericin T (Identity 22%)	Gassericin T (Identity 44%)	Gassericin T (Identity 44%)
Lysin	Listeriolysin (Identity 37%)	Listeriolysin (Identity 25%)	Listeriolysin (Identity 25%)

In silico genome mining of the three *L. crispatus* isolates for the presence of pathogen inhibition functions revealed several regions encoding genes for biosynthetic gene clusters (BGCs). All the three strains were identified to possess Bacteriocin_IIc which shared 22–44% identity to Gassericin T of *L. gasseri* and Lysin that shared identity to Listeriolysin (Supplementary Fig. 1C).

### Metabolic functionality uncovers key genes driving adaptability and stress resistance

Genome analysis by GutSMASH identified that all the three strains of *L. crispatus* possess the arginine deiminase (ADI) pathway that converts L‐arginine into L‐ornithine in anaerobic conditions. The arginine deiminase system functions as a source of energy by generating one molecule of ATP and also helps the bacteria to survive acidic environments. The strains harbor genes encoding lactate dehydrogenase (LDH), an enzyme responsible for lactic acid production, which lowers vaginal pH and mediates antimicrobial activity against opportunistic pathogens. In silico analysis of the biosynthetic capabilities of the strains revealed their ability to synthesize vitamins such as thiamine, riboflavin, retinol, B6 and folate. They also encoded genes for bile hydrolysis and taurine and hypotaurine metabolism pathways. Several key genes involved in short chain fatty acid synthesis were also detected in the three strains (Supplementary Table 2). All three strains lacked major metabolic pathways involved in the conversion of amino acids to biogenic amines with no genes involved in histidine decarboxylase pathway detected. Three genes each linked to tyrosine decarboxylase (*araT, gabD, adhE*) and lysine decarboxylase (*araT, gabD, atoB*) were detected. However, these genes are only involved in the upstream pathway of conversion of aromatic amino acids to aroma compounds and are not linked to biogenic amine production. Notably, *speA* gene which encodes for arginine decarboxylase (ADC) that converts arginine to agmatine was detected in all the three strains. However, the genes (*aguA, aguB*) responsible for agmatine metabolism to putrescine were absent in the strains highlighting their safety.

### In vitro antibacterial activity of *Lactobacillus crispatus* reveals resistance against Gram-negative and Gram-positive pathogens

**Fig. 1:**
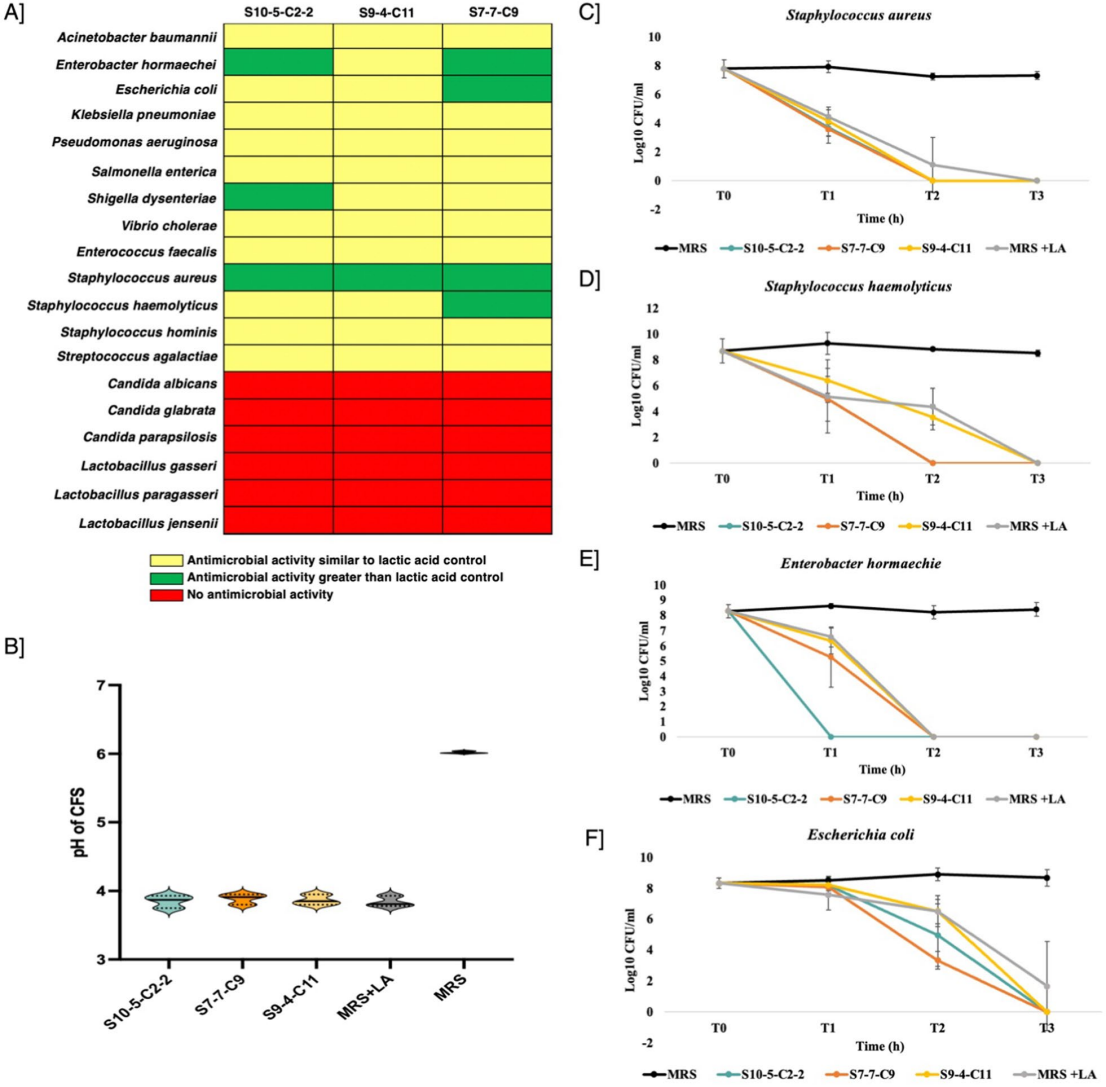
Antimicrobial activity of the cell free supernatants (CFS) of the *L. crispatus* strains. **A** Antimicrobial activity of *L. crispatus* CFS against a panel of bacterial and fungal pathogens and commensals. **B** pH range of the tested *L. crispatus* CFS in comparison with MRS broth. **C**–**F** Time- kill assay of *L. crispatus* CFS against selected Gram-negative and Gram-positive bacterial pathogens

We had previously demonstrated that the cell-free culture supernatant (CFS) of *L. crispatus* strains S10-5-C2-2, S9-4-C11 and S7-7-C9 effectively inhibited the growth of *Gardnerella vaginalis* ATCC 14018*, Escherichia coli* ATCC 25922 and *Klebsiella pneumoniae* ATCC 1705 [12]*.* Following these findings, we expanded our investigation to evaluate the antagonistic activity of the selected three *L. crispatus* strains on a broader range of Gram-negative and Gram-positive bacterial pathogens, fungal pathogens as well as commensal bacteria using the well diffusion assay. To distinguish whether the observed inhibition was due to acidic environment or other antimicrobial factors, MRS broth adjusted to the same pH as that of the CFS was included as a control. Figure 1A represents a heatmap summarizing the antimicrobial activity of CFS against each pathogen tested. In this representation, red indicates no antimicrobial activity, yellow indicates that the zone of inhibition produced by the CFS is equal to the MRS + LA control and the green indicates that the zone of inhibition produced by the CFS is larger than the MRS + LA control suggesting involvement of other antimicrobial factors such as the bacteriocins. The results revealed that the CFS of *L. crispatus* S10-5-C2-2 exhibited increased zone of inhibition as compared to the control against Gram-negative bacteria *Enterobacter hormaechei, Shigella dysenteriae* and Gram-positive bacteria *Staphylococcus aureus.* Similarly, *L. crispatus* S7-7-C9 demonstrated higher inhibitory activity against *Escherichia coli*, *E. hormaechei, S.aureus, Staphylococcus haemolyticus* and the S9-4-C11 strain against *S. aureus* likely mediated by the production of antimicrobial peptides*.* Moreover, the CFS of all three *L. crispatus* strains demonstrated zone of inhibition against a broad spectrum of Gram-negative and Gram-positive bacterial pathogens such as *Acinetobacter baumannii*, *Klebsiella pneumoniae, Salmonella enterica, Pseudomonas aeruginosa, Vibrio cholerae, Staphylococcus hominis, Streptococcus agalactiae* and *Enterococcus faecalis* comparable to the MRS + LA control. This antimicrobial effect was primarily attributed to the acidic environment of the CFS created by the lactic acid. In contrast, the CFS did not inhibit the growth of beneficial commensal bacteria, such as *Lactobacillus gasseri, Lactobacillus paragasseri* and *Lactobacillus jensenii* or any of the tested fungal pathogens (Fig. 1A). The pH of CFS from the three *L. crispatus* strains ranged between 3.7 and 3.95 while the pH of the MRS culture medium was near neutral (pH 6.02 ± 0.2) (Fig. 1B). Further, to validate the observation of well diffusion assay, time kill assays were performed against selected Gram-negative and Gram-positive pathogens for which the CFS showed maximum efficacy demonstrated that direct incubation of pathogens at 10^7^–10^8^ CFU/ml with *L. crispatus* CFS resulted in complete growth inhibition within 3 h (Fig. 1C–F). The effect was particularly pronounced against *S. aureus* with all three *L. crispatus* CFS inducing a 4 log₁₀ reduction in CFU/mL within just 1 h (Fig. 1C). Similarly, a 4 log₁₀ reduction of *S. haemolyticus* was observed within the first hour following incubation with S7-7-C9 CFS (Fig. 1D). Notably, *E. hormaechei* exhibited complete growth inhibition as early as 1 h when exposed to S10-5-C2-2 CFS (Fig. 1E). In contrast, all three *L. crispatus* CFS initially displayed no significant antibacterial activity against *E. coli* during the first hour. However, the CFS from S7-7-C9 achieved a 4 log₁₀ reduction in *E. coli* CFU/mL by 2 h. By 3 h, all three *L. crispatus* CFS could exert complete antimicrobial activity against *E. coli*, comparable to their effects on the other tested pathogens (Fig. 1F).

### In vitro probiotic characterization reveals gastric pH and bile salt tolerance of *Lactobacillus crispatus* strains

**Fig. 2:**
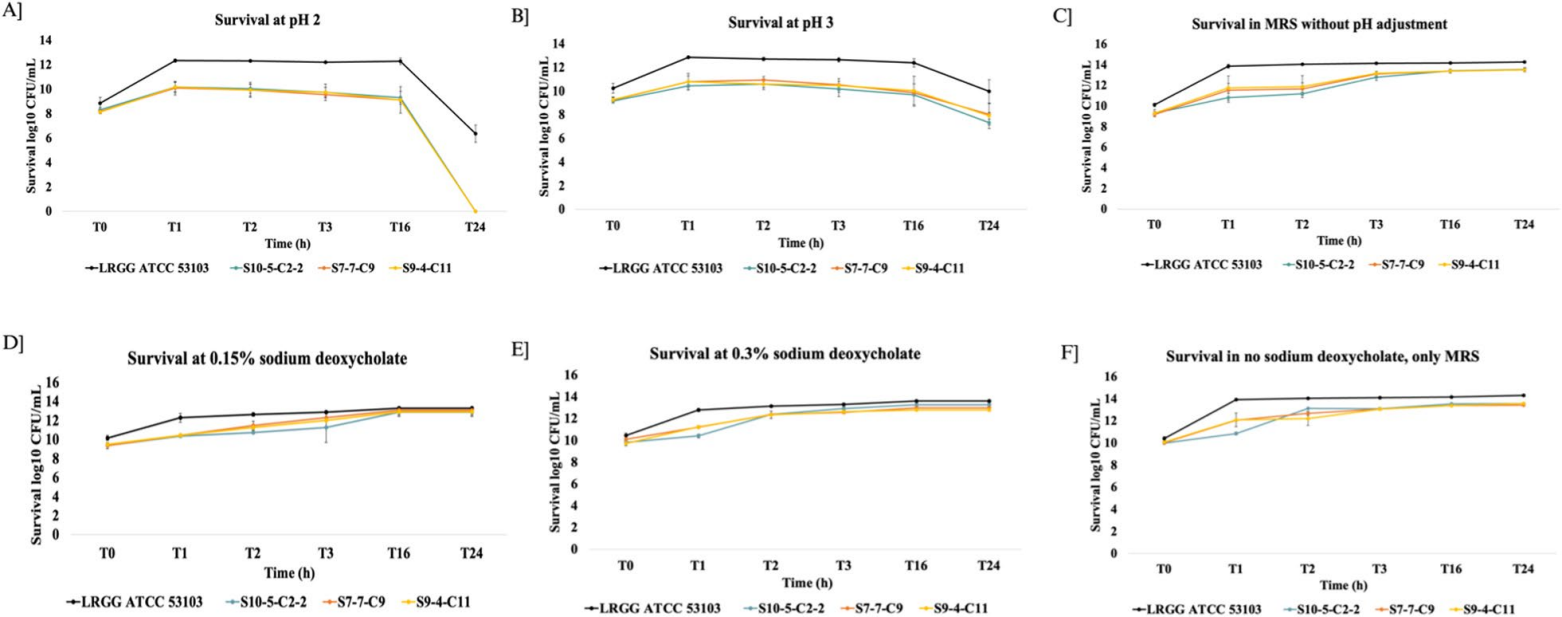
*L. crispatus* strains exhibit robust gastric pH and bile salt tolerance. **A** Survival of the *L. crispatus* strains in pH 2. **B** Survival of the *L. crispatus* strains in pH 3. **C** Survival of the *L. crispatus* strains in MRS without pH adjustment. **D** Survival of the *L. crispatus* strains in bile salt concentration 0.15%. **E** Survival of the *L. crispatus* strains in bile salt concentration 0.3%. **F** Survival of the *L. crispatus* strains in MRS without bile salt

The human stomach has a highly acidic environment typically with a pH between 2.0 and 3.5. This acidic milieu plays a critical role in the digestive process and serves as a primary barrier against ingested pathogens. For orally administered probiotic strains to exert their beneficial effects, one of the fundamental prerequisites is their ability to survive the harsh gastric environment and transit to the intestine, where they can colonize and confer health benefits to the host. Additionally, on reaching the intestine, probiotic bacteria are further challenged by the bile salts which are amphipathic, detergent-like molecules. After synthesized by the liver, bile salts are stored and secreted by the gall bladder to digest fats and lipids in food. These bile salts can also solubilize bacterial lipids and disrupt bacterial cell membranes, posing a significant threat to the bacterial viability. Therefore, bile-tolerance is an essential characteristic for any probiotic strains intended for gastrointestinal application. As a preliminary experiment before the in vivo studies, the ability of *L. crispatus* isolates to surpass the acidic environment of the stomach and survive in small intestine was assessed. Specifically, MRS broth adjusted to physiological gastric pH 2 and 3 and MRS broth containing bile concentrations 0.15% and 0.3% were used. All the three *L. crispatus* strains demonstrated robust pH tolerance with no decline in viability up to 16 h ensuring that the strains can transit through the acidic environment of the stomach and reach into the intestine (Fig. 2A–C). Furthermore, evaluation of the bile salt tolerance of the selected *L. crispatus* strains demonstrated that the strains retained complete viability and could survive sodium deoxycholate concentrations up to 0.3% till 24 h indicating strong resistance to bile-induced stress (Fig. 2D–F). Collectively, these findings highlight that the *L. crispatus* strains S10-5-C2-2, S9-4-C11 and S7-7-C9 possess strong in vitro probiotic traits, specifically, tolerance to gastric acidity and bile salts making them suitable candidates for oral administration, which remains the most economical and practical route for probiotic delivery.

### Lyophilized *Lactobacillus crispatus* maintains viability and storage stability

**Fig. 3:**
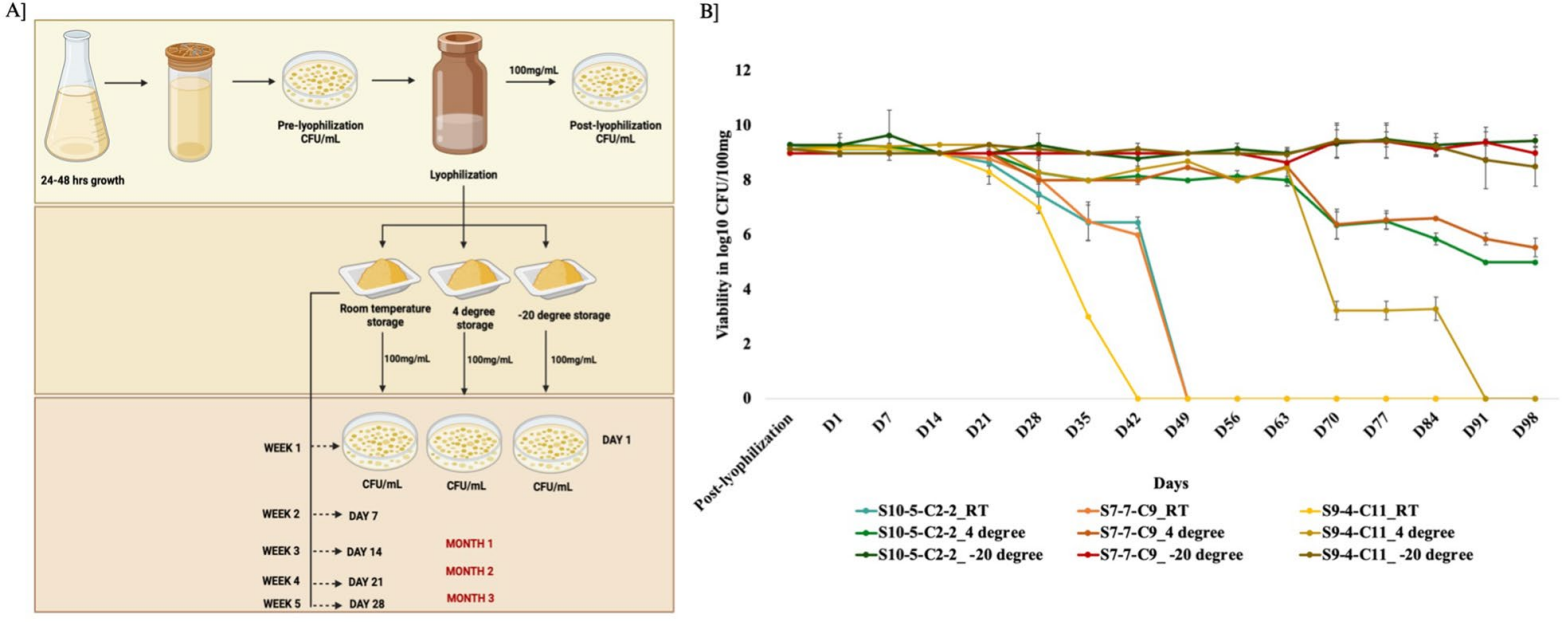
Viability of lyophilized *L. crispatus* strains S10-5-C2-2, S9-4-C11 and S7-7-C9. **A** Schematic representation of lyophilization and viability testing. **B** Survival (log10 CFU/100 mg) of lyophilized *L. crispatus* strains S10-5-C2-2, S9-4-C11 and S7-7-C9 stored at room temperature, 4 °C and − 20 °C

The *L. crispatus* strains lyophilized using sucrose (10% w/v) as a cryoprotectant remained viable during long-term storage under controlled temperature conditions. Schematics of viability assay is represented in Fig. 3A. Viability assays conducted over a three months period demonstrated that bacterial counts were stably maintained when stored at 4 °C and − 20 °C, with minimal loss in CFUs (Fig. 3B). No significant decline in viability was observed across time points, indicating robust long-term stability in freezer conditions. All three *L. crispatus* strains maintained 100% viability at − 20 °C throughout the study. Storage at 4 °C resulted in a 40% reduction in viability for strains S10-5-C2-2 and S7-7-C9 strains. However, the strain S9-4-C11 was less stable at higher temperatures of RT and 4 °C with it losing viability at day 42 and 91 respectively. Nevertheless, the preservation of the lyophilized strains and maintenance of its viability under cold storage conditions (− 20 °C) enabled consistent dosing during the animal experiments and ensured reproducibility of results throughout the study duration. The demonstrated stability supports the feasibility of developing *L. crispatus*-based biotherapeutic products, particularly where cold-chain logistics or extended shelf life are required.

### *L. crispatus* attenuated body weight gain, visceral fat and insulin resistance in CDHF fed mice

**Fig. 4:**
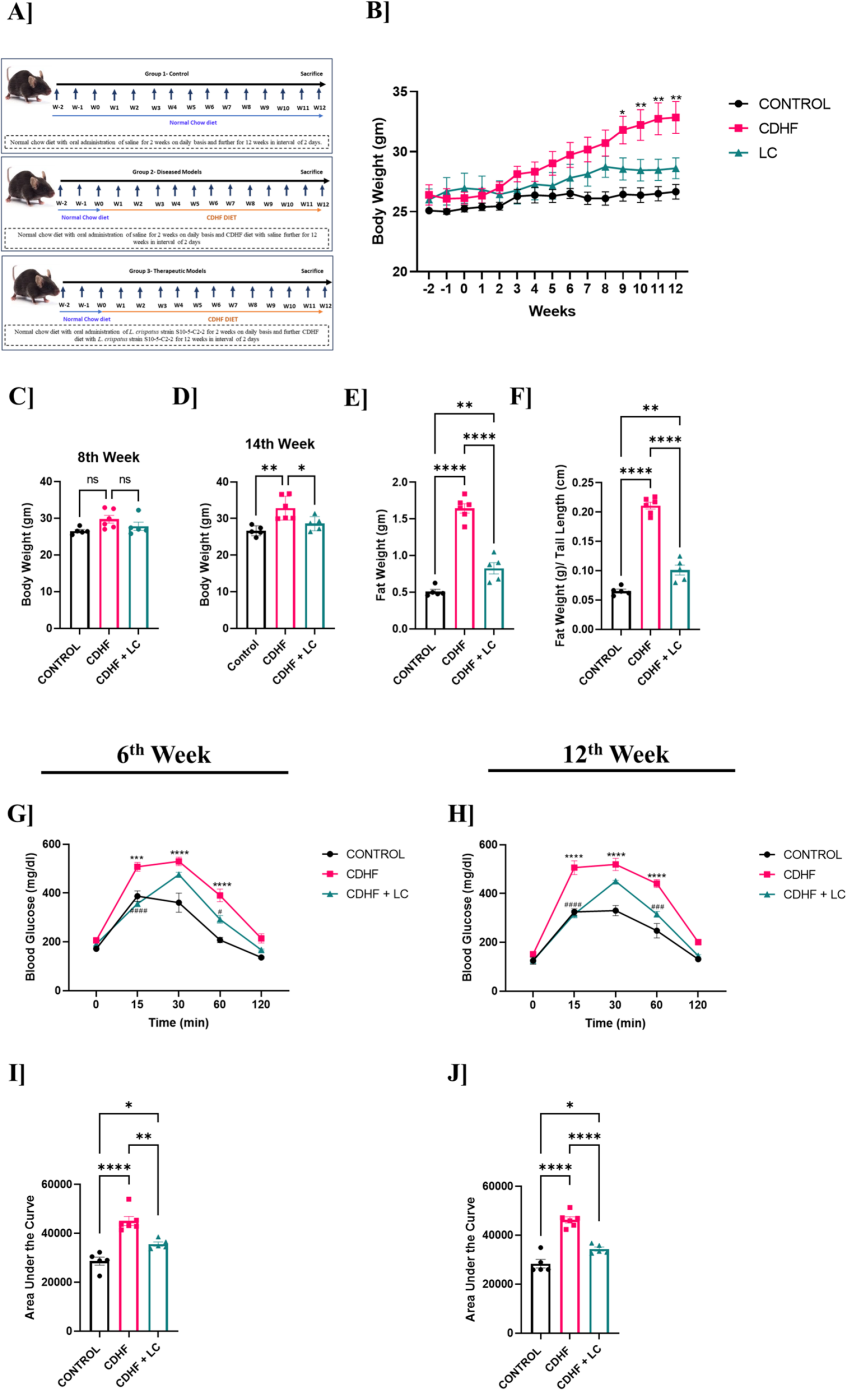
Graphs representing the Body weight, Fat weight and Intra peritoneal glucose tolerant test (IPGTT) and its area under the curve data of Control, CDHF, and CDHF + *L. crispatus* (LC) mice groups at 6th and 12th weeks respectively. **A** Animal Study Design **B** Weekly body weight analysis **C** 8th week body weight **D** 12th week body weight **E** Fat weight **F** Fat weight by Tail length **G**, **H** IPGTT graphs and **I**, **J** bar graphs showing the area under the curve of IPGTT. Values are represented as Mean ± SEM. Statistics was done by One-way ANOVA and Two-way ANOVA followed by Tukey's test where *p < 0.05, **p < 0.01, ***p < 0.001, ****p < 0.0001 N = 5–6

Since all three *L. crispatus* stains exhibited comparable genomic and in vitro probiotic characteristics, we chose one representative strain, *L. crispatus*, strain S10-5-C2-2 which demonstrated the most consistent performance across all assays, for further in vivo evaluation in a cardiometabolic disease mice model. The experimental design involved three groups of mice: a control group, a group fed with CDHF diet, and a group fed the CDHF diet and orally supplemented with *L. crispatus* S10-5-C2-2 (Fig. 4A). Weekly body weight measurements over 12 weeks revealed a significant increase in weight in the CDHF-fed mice compared to controls, with the effect becoming evident from the fourth week onward. Supplementation with *L. crispatus* effectively attenuated this weight gain throughout the study period (Fig. 4B). Further, a comparison of body weights at week 8 and week 12 confirmed that CDHF-fed mice had significantly higher body weights at both time points compared to the control group, while *L. crispatus*-supplemented mice exhibited notably reduced body weight compared to the group fed with CDHF diet only (Fig. 4C and D). Analysis of adiposity revealed that the CDHF diet significantly increased visceral fat accumulation compared to the control group (Fig. 1E). This fat gain was significantly mitigated in mice receiving *L. crispatus* supplementation. Additionally, normalization of fat weight to tail length demonstrated a similar pattern, with *L. crispatus*-treated mice showing reduced fat accumulation relative to the CDHF group (Fig. 4F). To evaluate glucose metabolism, intraperitoneal glucose tolerance tests (IPGTT) were conducted at weeks 6 and 12. At week 6, CDHF-fed mice displayed marked glucose intolerance, characterized by elevated blood glucose levels and higher area under the curve (AUC) values compared to controls. In contrast, *L. crispatus* supplementation resulted in improved glucose clearance and significantly reduced AUC (Fig. 4G and I). This improvement was sustained at week 12, where *L. crispatus*-treated mice continued to exhibit improved glucose handling and significantly lower AUC values relative to the CDHF-fed group (Fig. 4H and J). Together, these findings demonstrate that *L. crispatus* supplementation mitigates CDHF-induced weight gain, adiposity, and glucose intolerance.

### *Lactobacillus crispatus* reduce hepatic steatosis and fibrosis in mice fed with CDHF diet

**Fig. 5:**
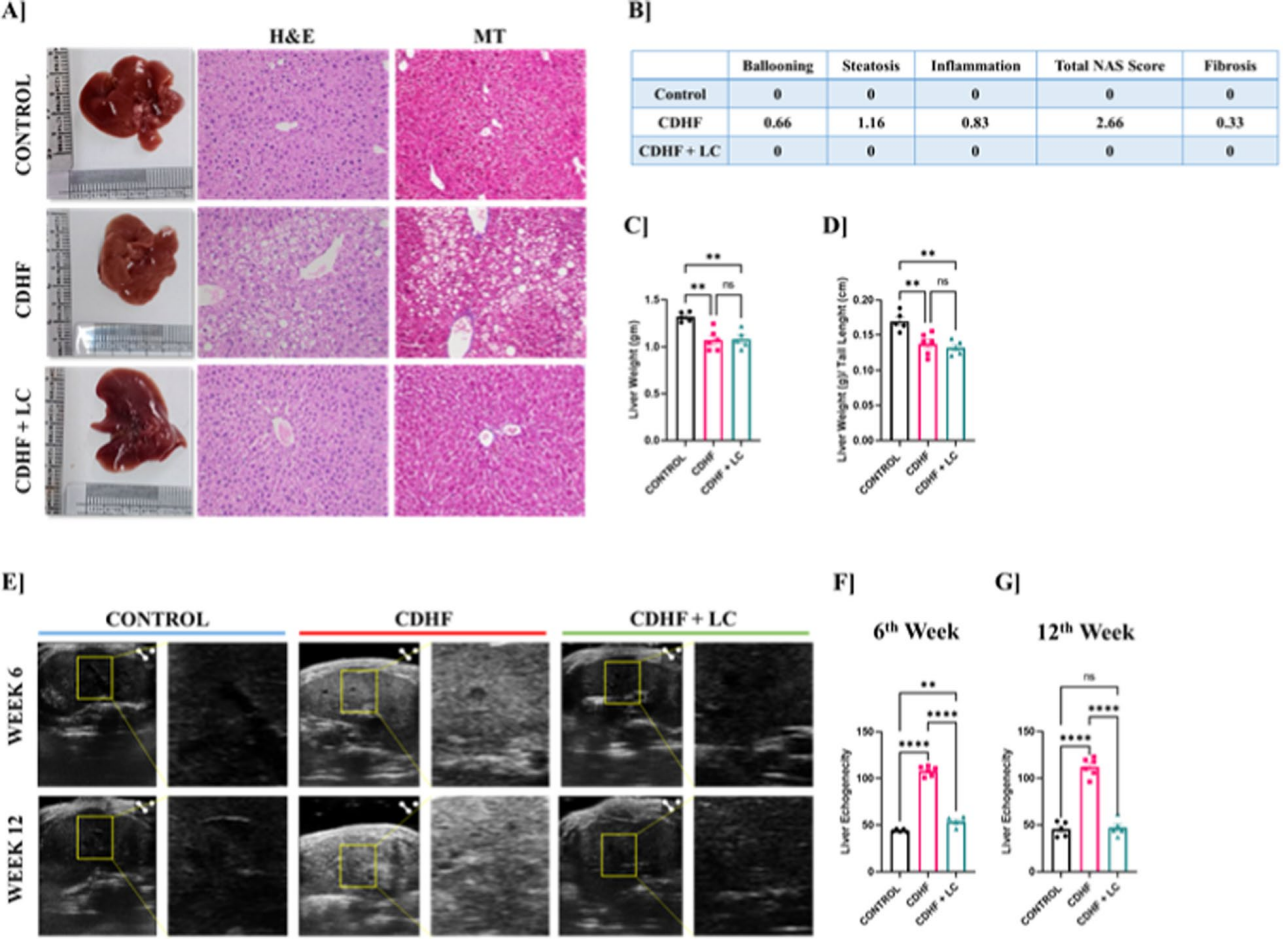
Liver histopathology with NAS scoring and echogenicity of the liver by ultrasound among Control, CDHF, and CDHF + LC mice groups at 6th and 12th weeks. **A** Histopathology of the liver. Light micrograph of the liver sections from different groups (20X) stained with hematoxylin and eosin and Masson's Trichrome **B** NAFLD activity score representing steatosis, ballooning and inflammation **C** Liver weight **D** Liver weight by Tail length **E** Echogenicity ultrasound B mode images **F** 6th week echogenicity score bar graph **G** 12th week echogenicity score bar graph. Values are represented as Mean ± SEM. Statistics was done by One-way ANOVA followed by Tukey's test where *p < 0.05, **p < 0.01, ***p < 0.001, ****p < 0.0001 N = 5–6

At the completion of the 12-week intervention, gross morphological assessment of excised livers revealed visible differences among the groups (Fig. 5A). The control group exhibited smooth, uniformly colored liver surfaces, while livers from the CDHF-fed mice appeared pale, irregular, and enlarged. Remarkably, the livers from the *L. crispatus* supplemented group showed improved morphology with reduced pallor and irregularity. Histopathological examination using hematoxylin and eosin (H&E) staining also confirmed these observations, with CDHF-fed mice displaying extensive lipid droplet accumulation and hepatic ballooning (Fig. 5A). In contrast, *L. crispatus* supplementation notably reduced hepatic fat accumulation and cellular degeneration. Fibrosis was further evaluated using Masson's trichrome staining, which revealed increased collagen deposition in the CDHF group, while minimal or no fibrosis was observed in the control and *L. crispatus*-treated groups. Quantitative scoring based on the NAFLD Activity Score (NAS) showed a significant increase in steatosis, lobular inflammation, and ballooning in the CDHF-fed group, while the *L. crispatus*-treated group exhibited a marked reduction in all parameters, indicating histological improvement (Fig. 5B). In terms of liver weight, CDHF-fed mice demonstrated significantly decreased liver mass compared to controls (Fig. 5C), with normalization to tail length further confirming significant decrease in the liver weight (Fig. 5D). *L. crispatus* supplementation does not show significant change in liver weight. To non-invasively assess hepatic steatosis progression, echogenicity was measured using ultrasound imaging at both 6 and 12 weeks (Fig. 5E). CDHF-fed mice exhibited elevated hepatic echogenicity indicative of fat accumulation, whereas mice receiving *L. crispatus* showed reduced echogenicity, suggesting an amelioration of hepatic steatosis. Quantitative analysis revealed significantly higher echogenicity scores in the CDHF group at both 6 and 12 weeks, which were substantially decreased upon *L. crispatus* treatment (Fig. 5F and G). These findings collectively demonstrate that *L. crispatus* supplementation mitigates diet-induced hepatic lipid accumulation, inflammation, fibrosis, and steatosis progression, as evidenced by both histological and imaging-based assessments.

### *Lactobacillus crispatus* attenuates cardiac inflammation and fibrosis in CDHF diet-induced mice

**Fig. 6:**
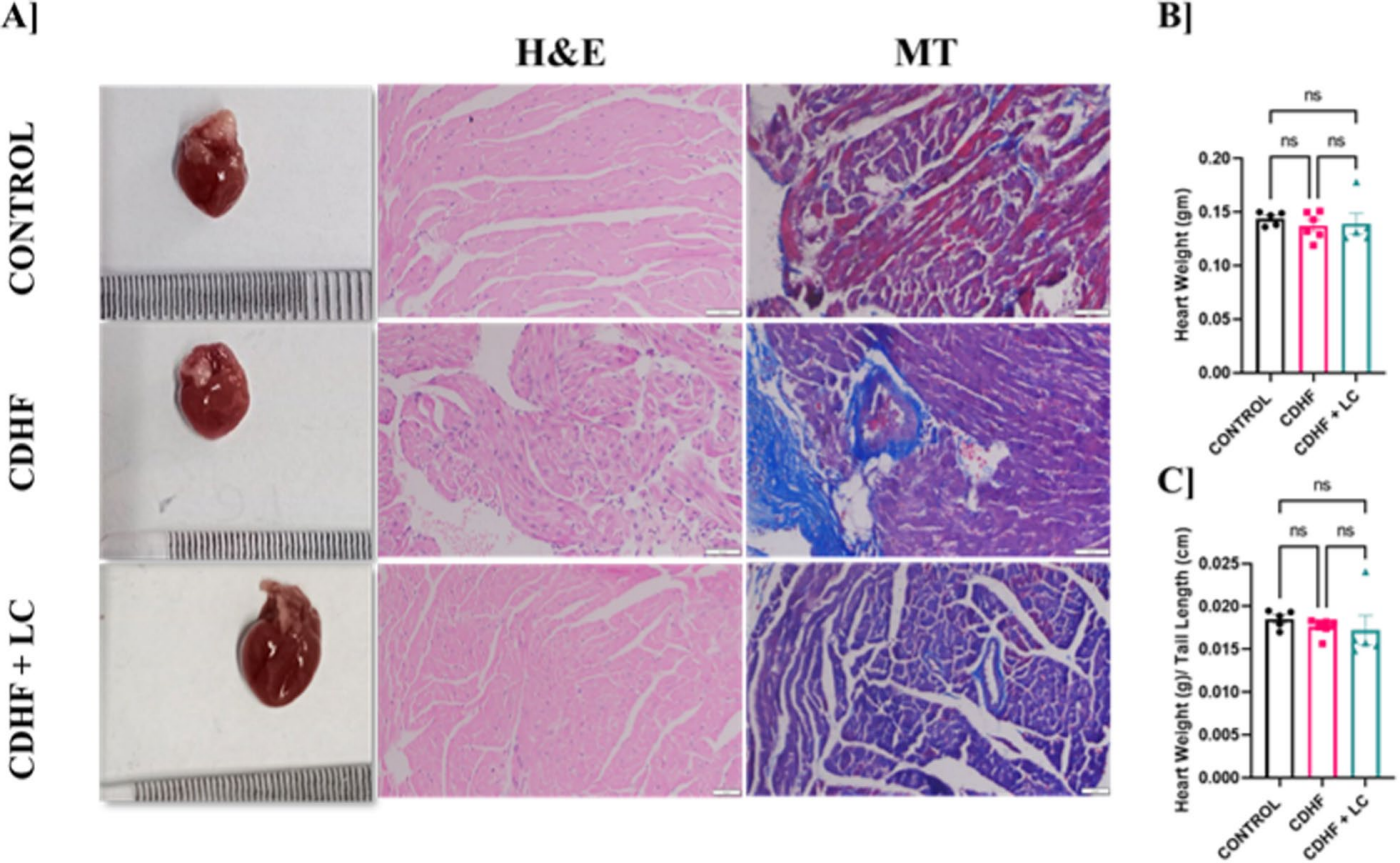
Heart histopathology and heart weight comparison among Control, CDHF, and CDHF + LC mice groups at 12th weeks. **A** Histopathology of the heart. Light micrograph of the heart sections from different groups (20X) stained with hematoxylin and eosin and Masson's Trichrome **B** Heart weight **C** Heart weight by Tail length. Values are represented as Mean ± SEM. Statistics was done by One-way ANOVA followed by Tukey's test where *p < 0.05, **p < 0.01, ***p < 0.001, ****p < 0.0001 N = 5–6

To assess the impact of *L. crispatus* supplementation on cardiac morphology and histopathology on the CDHF diet-fed mice, heart tissues were collected at the end of the 12-week experimental period. Gross examination revealed that the hearts of all the groups have similar morphology of heart (Fig. 6A). Histopathological analysis using hematoxylin and eosin (H&E) staining demonstrated that CDHF feeding induced disorganization of cardiomyocyte structure, with evidence of myocyte swelling and interstitial expansion. Further, increased in the neutrophil counts in heart tissue sections indicates presence of cardiac inflammation in CDHF-fed mouse. In contrast, cardiac tissue from the *L. crispatus*-treated group maintained better cellular architecture with reduced signs of structural damage and cardiac inflammation. Masson's trichrome staining revealed significant collagen deposition and fibrotic remodeling in the myocardium of CDHF-fed mice, indicative of cardiac fibrosis. Notably, *L. crispatus* supplementation mitigated this fibrotic response, with minimal collagen accumulation observed, comparable to the control group. Although there are no changes observed in the ‘heart weight’ and ‘heart weight by tail length ratio’ (Fig. 6B). These data collectively suggest that CDHF diet induces cardiac inflammation and fibrosis, while *L. crispatus* supplementation ameliorates these pathological alterations, preserving cardiac structure and function.

### *Lactobacillus crispatus* improved cardiac functional parameter measured by echocardiography in mice fed with CDHF diet

**Fig. 7:**
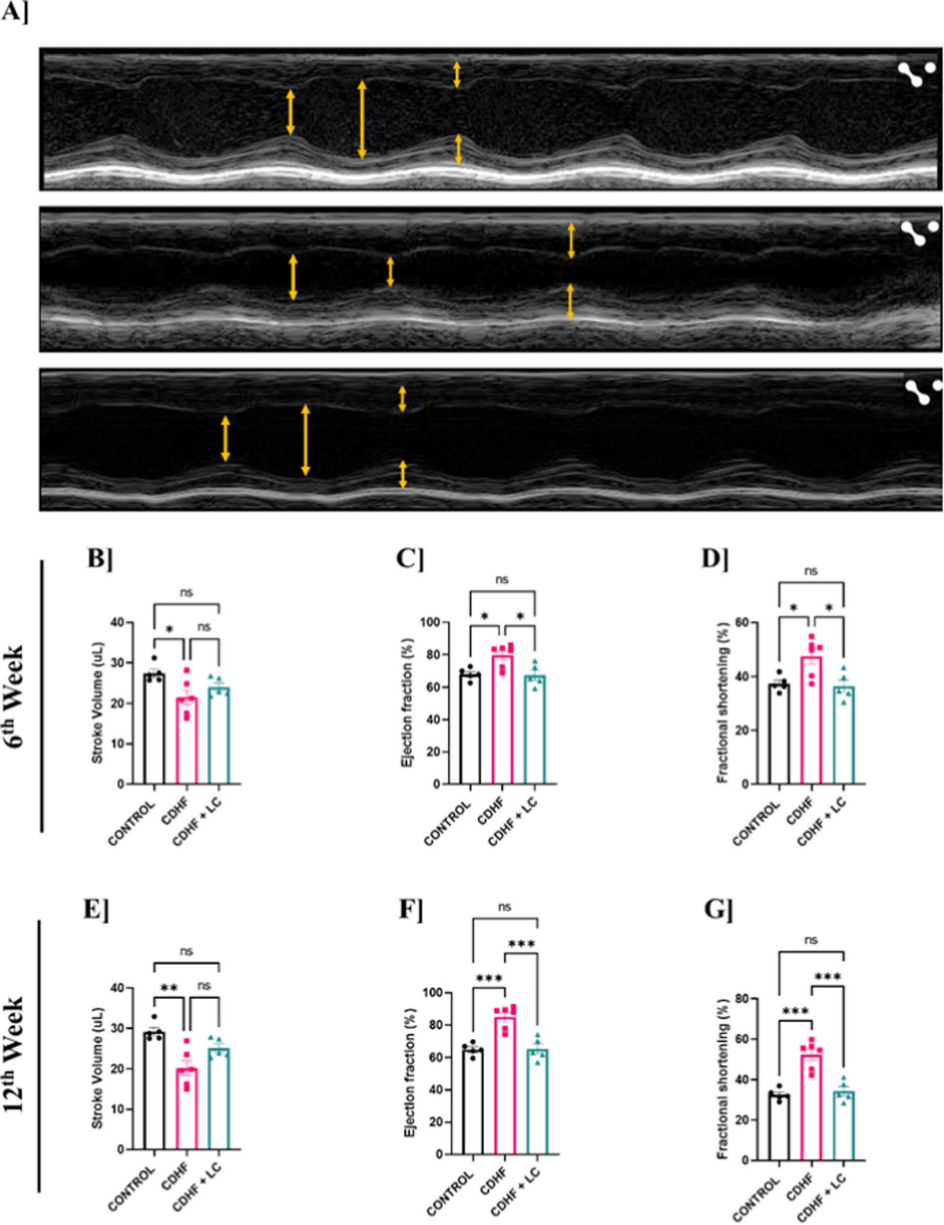
Changes in functional parameters of the heart measured by echocardiography at 6th and 12th week among Control, CDHF and CDHF + LC groups. **A** M-mode echocardiography of heart **B** 6th week stroke volume **C** 6th week ejection fraction **D** 6th week fractional shortening **E** 12th week stroke volume **F** 12th week ejection fraction **G** 12th week fractional shortening. Values are represented as Mean ± SEM. Statistics was done by One-way ANOVA followed by Tukey's test where *p < 0.05, **p < 0.01, ***p < 0.001, ****p < 0.0001 N = 5–6

In the present study, we measured cardiac function by echocardiography in control, CDHF and CDHF + *L. crispatus* mice at 6th and 12th week of the study. Transthoracic echocardiography was performed with a 15 MHz linear array transducer system. Mice were anesthetized with isoflurane (2%) in anesthesia chamber and the chest hair was removed. The transducer was placed on the left hemothorax. Two dimensionally guided left ventricle (LV) M-mode images at the papillary muscle level were obtained from the parasternal short axis view. 2D echocardiography images were used to measure structural as well as functional parameters of hearts (Fig. 7A).

The echocardiography data (Fig. 7B–G) has shown a significant increase in ejection fraction and fractional shortening in CDHF mice in 6th week and 12th weeks. Further, *L. crispatus* has significantly downregulated ejection fraction and fractional shortening in both time points in CDHF + *L. crispatus* group. In the present study, an increased fractional shortening indicates that the ventricle is contracting more. Further, a decrease in stroke volume was observed at 6th and 12th week of CDHF group compared to control. However, *L. crispatus* treatment showed the trend of increasing the stroke volume in 6th and 12th week in CDHF + *L. crispatus* mice but it is not significant change.

### *Lactobacillus crispatus* improved cardiac left ventricular (LV) mass and wall thickness parameters in CDHF fed mice

**Fig. 8:**
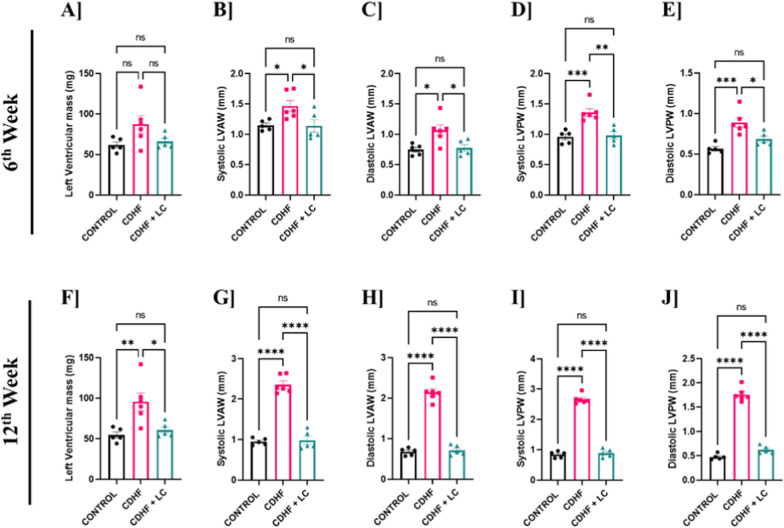
Changes in LV mass and cardiac wall thickness of heart measured by echocardiography at 6th and 12th week of Control, CDHF and CDHF + LC. **A** 6th week left ventricular mass **B** 6th week systolic LVAW **C** 6th week diastolic LVAW **D** 6th week systolic LVPW **E** 6th week diastolic LVPW **F** 12th week left ventricular mass **G** 12th week systolic LVAW **H** 12th week diastolic LVAW **I** 12th week systolic LVPW **J** 12th week diastolic LVPW. Values are represented as Mean ± SEM. Statistics was done by One-way ANOVA followed by Tukey's test where *p < 0.05, **p < 0.01, ***p < 0.001, ****p < 0.0001 N = 5–6

In the present study, we further evaluated structural changes in cardiac morphology, focusing on ventricular wall thickness as an early indicator of cardiac remodeling and hypertrophy. Specifically, we measured the left ventricular anterior wall thickness (LVAW) and posterior wall thickness (LVPW) during both systole (s) and diastole (d) (LVAW; s, LVAW; d, LVPW; s, and LVPW; d) at 6th and 12th weeks of intervention (Fig. 8A–J). Our findings demonstrated a significant increase in all these parameters in the CDHF-fed group compared to the control group at both time points, indicating the presence of ventricular wall thickening. This thickening reflects alterations in both systolic and diastolic phases of left ventricular function, suggesting the onset of concentric hypertrophy likely as an adaptive response to the metabolic stress induced by the CDHF diet. Importantly, mice receiving *L. crispatus* supplementation alongside the CDHF diet showed a marked reversal of these pathological changes. At week 6, the *L. crispatus*-treated group exhibited significantly lower values of LVAW and LVPW thickness compared to the untreated CDHF group, suggesting a protective effect of *L. crispatus* against early cardiac remodeling. Notably, this improvement was even more pronounced by the 12th week, with ventricular wall measurements in the CDHF + *L. crispatus* group approaching values of the control animals. These findings indicate that *L. crispatus* not only prevents but also progressively reverse left ventricular hypertrophy induced by dietary and metabolic stress, with the effects becoming more robust over time.

Further the diameter and volume of systole and diastole was observed till 12th week (Figure S2). At 6th and 12th week, a significant decrease in systolic volume and diameter, diastolic diameter and volume was observed in CDHF fed mice*.* At 6th week, *L. crispatus* significantly increased the systolic volume and diameter in the CDHF + *L. crispatus* group but not showed any significant increase in the diastolic diameter and volume. Further, in the 12th week all the parameters are significantly recovered by the *L. crispatus* when compared to CDHF fed mice.

### Impact of *Lactobacillus crispatus* treatment on serum metabolite profiles in CDHF diet-fed mice

**Fig. 9:**
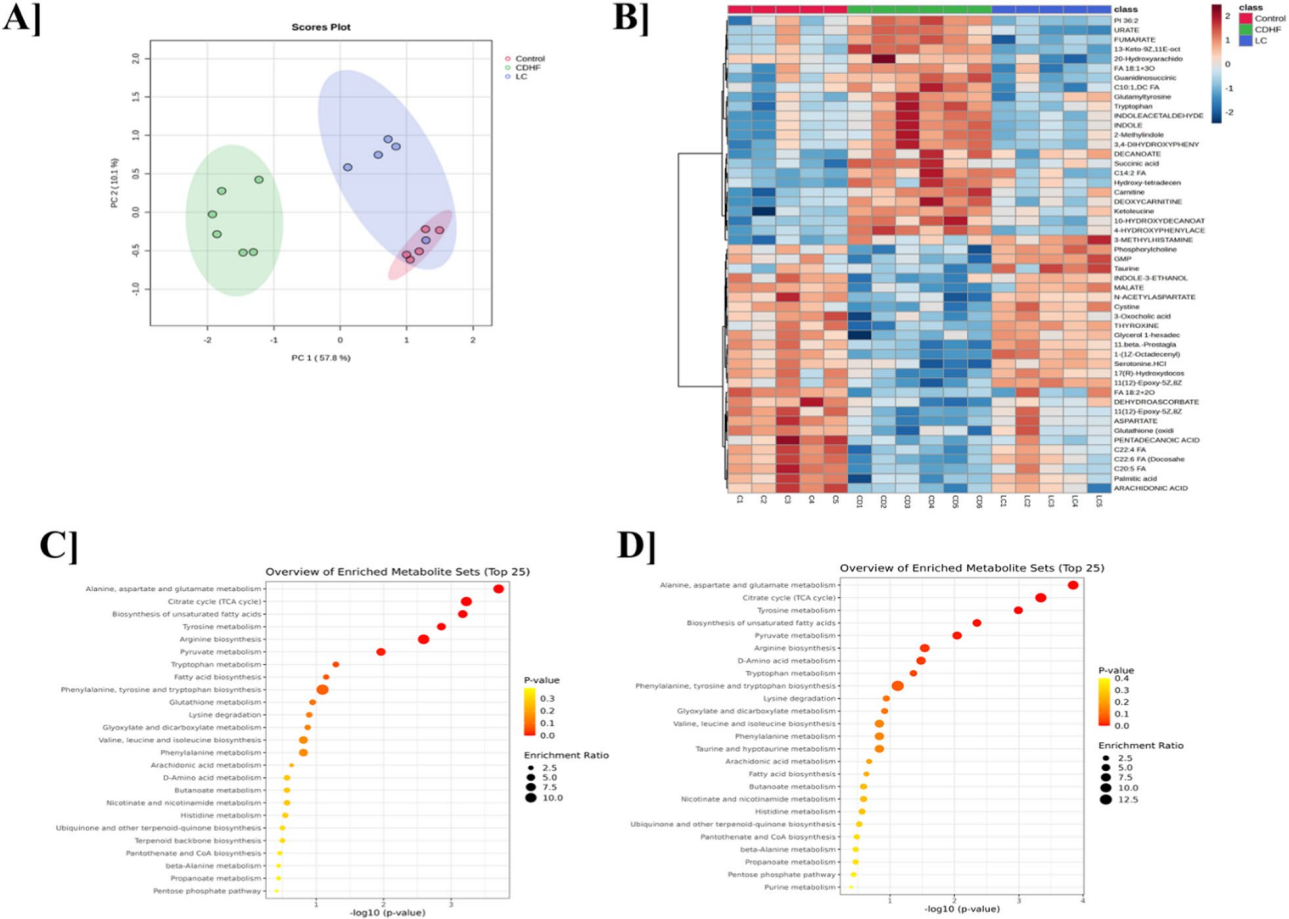
Serum metabolomics alteration after CDHF diet and *L. crispatus* treatment. **A** PCA scores plots of metabolites. Dynamic distribution of metabolite content differences. **B** Heatmap demonstrating the top 50 serum metabolites differentiating control, CDHF and CDHF + LC by one way ANOVA analysis. Metabolic analysis is based on the KEGG database, and enriched pathways are displayed by bubble plots **C** Control vs CDHF and **D** CDHF vs *L. crispatus*

To evaluate the metabolic alterations induced by the CDHF diet and the modulatory effect of *L. crispatus* supplementation, untargeted metabolomics analysis was performed on serum samples from all experimental groups. Principal component analysis (PCA) revealed distinct clustering patterns among the control, CDHF, and CDHF + *L. crispatus* groups (Fig. 9A). The control group clustered separately from CDHF and *L. crispatus*-treated groups, indicating substantial metabolic shifts in response to dietary intervention. Notably, the *L. crispatus* group showed partial separation from the CDHF group and a closer proximity toward the control cluster, suggesting that *L. crispatus* modulates the systemic metabolite profile toward a more normalized state. A comprehensive heat map analysis of differential metabolites further confirmed these findings (Fig. 9B). The CDHF group exhibited significant alterations in multiple metabolite classes, with widespread dysregulation in amino acid metabolism, lipid metabolism, and energy-related pathways. Specifically, metabolites associated with fatty acid biosynthesis were upregulated in the CDHF group, while metabolites involved in fatty acid β-oxidation were markedly downregulated, indicating an imbalance in lipid utilization and storage. In contrast, *L. crispatus* treatment reversed many of these changes, with a visible normalization of both upregulated and downregulated metabolites, particularly those linked to lipid metabolism. To gain functional insights into the affected pathways, metabolite set enrichment analysis was performed. In the comparison between control and CDHF groups (Fig. 9C), pathways such as fatty acid biosynthesis and arachidonic acid metabolism were significantly enriched, reflecting increased lipid accumulation and inflammatory potential. Furthermore, there was a clear suppression of fatty acid β-oxidation, suggesting impaired mitochondrial lipid catabolism. In contrast, comparison between the CDHF and CDHF + *L. crispatus* groups (Fig. 9D) revealed that *L. crispatus* supplementation significantly modulated both fatty acid biosynthesis and β-oxidation pathways. While the enrichment of fatty acid biosynthesis was reduced, markers of fatty acid β-oxidation were upregulated, indicating a shift from lipogenesis toward improved lipid oxidation and energy production in *L. crispatus* group.

### *L. crispatus* inhibit hepatic lipogenesis by activating AMPK signaling

**Fig. 10:**
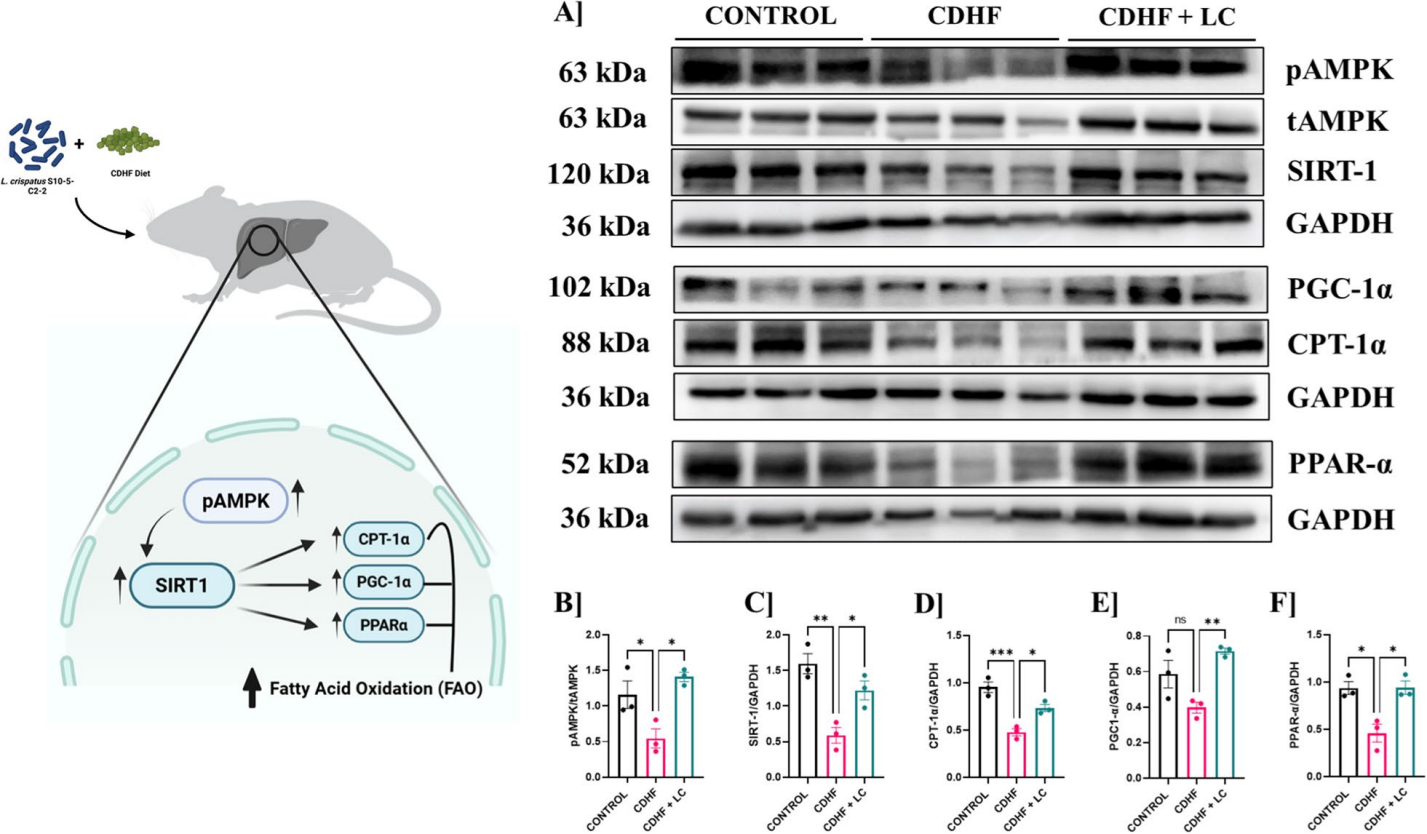
*L. crispatus* inhibits hepatic fatty acid synthesis-associated protein and gene expression in CDHF-administered mice. **A** Expression of proteins related to energy metabolism, β-oxidation of fatty acids and mitochondria in the liver was assessed by immune blot; **B** pAMPK/tAMPK expression; **C** SIRT-1/GAPDH; **D** CPT-1α/GAPDH; **E** PGC-1α/GAPDH; **F** PPAR-α/GAPDH. Values are represented as Mean ± SEM. Statistics was done by One-way ANOVA followed by Tukey's test where *p < 0.05, ** p < 0.01, *** p <  0.001, N = 5-6

We explored the molecular basis of *L. crispatus*-mediated improvements in hepatic lipid metabolism in mice fed with CDHF diet. We examined the expression of key regulators involved in fatty acid oxidation through immunoblotting in liver tissues of control, CDHF, and CDHF + *L. crispatus* group mice. The results revealed that CDHF feeding led to a marked downregulation in the expression of phosphorylated AMP-activated protein kinase (pAMPK), sirtuin 1 (SIRT1), peroxisome proliferator-activated receptor gamma coactivator 1-alpha (PGC-1α), carnitine palmitoyltransferase 1 alpha (CPT-1α), and peroxisome proliferator-activated receptor alpha (PPARα), indicating a significant suppression of the AMPK-SIRT1-PPARα signaling axis, which plays a central role in mitochondrial biogenesis and β-oxidation of fatty acids (Fig. 10). This downregulation implies impaired energy sensing, reduced mitochondrial oxidative capacity, and hampered lipid clearance in the liver under chronic dietary fat exposure. Further, supplementation with *L. crispatus* substantially restored the expression of these proteins, suggesting a protective and regulatory effect on lipid homeostasis. Densitometric analysis of the immunoblots further confirmed the elevated expression of pAMPK and SIRT1 in the CDHF + *L. crispatus* group, supporting reactivation of cellular energy sensors and NAD⁺-dependent deacetylation processes that are essential for downstream metabolic regulation. The significant upregulation of PGC-1α and CPT-1α, two critical mediators of mitochondrial energy metabolism and fatty acid transport into the mitochondria, respectively, suggests enhanced mitochondrial functionality and fatty acid catabolic flux following probiotic intervention. Additionally, the observed elevation in PPARα levels further indicates transcriptional reprogramming toward increased fatty acid oxidation and decreased lipid accumulation. These findings mechanistically align with our metabolomic observations and collectively highlight that *L. crispatus* exerts its beneficial effects by modulating the hepatic AMPK-SIRT1-PGC-1α-PPARα-CPT1α pathway, thereby promoting mitochondrial fatty acid β-oxidation and mitigating the deleterious metabolic alterations induced by CDHF diet. This integrated molecular and functional evidence underscores the therapeutic potential of *L. crispatus* in restoring metabolic flexibility and preventing lipid-driven hepatic dysfunction.

## Discussion

*L. crispatus* has long been recognized as a key biomarker of vaginal health and showed its protective effect against colonization of allochthonous microbiota [32]. The dominance of *L. crispatus* contributes to pathogen resistance primarily through the production of lactic acid and antimicrobial peptides, which helps maintain a low vaginal pH and inhibit the growth of opportunistic pathogens [33, 34]. Several well characterized *L. crispatus* strains have been developed into vaginal probiotics such as Crispact and LACTIN-V, and are in the final phases of clinical trials. However, their efficacy has been primarily studied only in reproductive health.

In our previous study, we isolated and performed whole genome sequencing of sixty *L. crispatus* strains from the vaginal microbiota of healthy Indian women enrolled in the GARBH-INi cohort. Comparative genomic analysis revealed that these *L. crispatus* strains harbored the highest number of biosynthetic gene clusters (BGCs) compared to other *Lactobacillus* species isolated from the vaginal microbiota, including *L. gasseri, L. paragasseri* and *L. jensenii* [35]. In the present study, broader health promoting potential of *L. crispatus* was explored, focusing on strain level characterization of selected candidate strains and translation for developing novel biotherapeutic for cardiometabolic disorders. Strain-level characterization is essential for delineating specific metabolic functions, evaluating safety and guiding therapeutic use. Accordingly, three *L. crispatus* strains harboring multiple genes linked to probiotic functionality as revealed from our previous study were evaluated further for additional genetic potential in biogenic amine biosynthesis, short chain fatty acid biosynthesis, vitamin biosynthesis and bile salt metabolism. Biogenic amines produced by bacterial isolates can elevate vaginal pH, thereby promoting the colonization of opportunistic pathogens. Moreover, excessive production of these amines upon ingestion may lead to adverse physiological effects such as headache, vomiting, diarrhea and heart palpitations [34]. Notably, none of the selected strains harbored key genes required for biogenic amine synthesis, underscoring their safety and potential for oral administration. Further, metabolic pathway reconstruction and functional profiling revealed that the *L. crispatus* possesses genes involved in bile salt metabolism, short chain fatty acid biosynthesis and vitamin biosynthesis, particularly folate, suggesting a role in modulating host metabolic health similar to traditional gut probiotics. Future metabolomics studies leveraging these strains would precisely quantify the production levels of these beneficial metabolites, further deepening our understanding of strain-specific metabolic capabilities. International regulatory bodies such as Food and Drug Administration (FDA) and World Health Organization (WHO) require that probiotics do not carry transferable AMR genes or any virulence factors. The presence of such genes in the probiotic bacteria poses a potential risk of horizontal gene transfer to commensal or pathogenic members of the host microbiota, which could compromise antibiotic treatment efficacy [35]. Tian et al. had previously reported presence of antimicrobial genes in probiotic bacteria derived from commercial health supplements and highlighted their transmission to bacteria such as *E. coli* and *S. aureus* [35]. In contrast, the *L. crispatus* strains analyzed in this study did not harbor any transferable resistance genes underscoring their genomic safety for probiotic use.

Importantly, cell-free culture supernatants from the *L. crispatus* strains demonstrated potent and selective antimicrobial activity against a panel of clinically relevant enteric, urinary and dermal pathogens while sparing commensal bacteria like another *Lactobacillus* spp. The cell-free culture supernatants exerted early bactericidal effects against Gram-negative bacteria, *E. coli* and *E. hormaechei,* as well as Gram-positive bacteria *S. aureus* and *S. haemolyticus.* Notably this activity was not replicated by MRS medium adjusted to similar pH, suggesting the involvement of additional bioactive molecules such as bacteriocins and lysins. Bacteriocins and lysins produced by the strains have been detected through LC–MS/MS and reported previously by our group. However, lactic acid production also contributed significantly in the antimicrobial efficacy against several pathogens tested indicating that both acidification and strain specific antimicrobials play complementary roles in pathogen inhibition. A similar broad-spectrum antimicrobial activity was previously reported by Santarelli et al. for *L. crispatus* LcM247, the probiotic strain used in commercial product Crispact® (Pharmextracta S.p.A., Piacenza, Italy) demonstrating inhibition against several common pathogenic bacteria [26].

Several studies have previously examined the gastric pH and bile tolerance of *L. crispatus* strains isolated from vaginal microbiota [36–38]. Notably, Abramov et al. and Anglenius et al. demonstrated that not all *L. crispatus* strains can successfully transit through the gut conditions to manifest the probiotic properties and there are marked strain-dependent variability in acid and bile tolerance [36, 37]. In the present study, prior to conducting in vivo experiments, we assessed the ability of the selected *L. crispatus* strains to survive the gastric pH (pH 2–3) and physiological bile salt concentrations to determine their resilience and potential for successful intestinal colonization. All the three strains demonstrated strong tolerance to gastric pH and bile stress, suggesting that they could be formulated for oral administration. Additionally, the strains remained viable under cold storage after lyophilization, indicating their suitability for large-scale formulation and long-term storage in probiotic preparations.

Further, this study provides compelling evidence that oral supplementation with *L. crispatus* effectively mitigates the systemic metabolic, hepatic, and cardiac pathologies induced by a choline-deficient high-fat (CDHF) diet in mice. The CDHF diet successfully induced a phenotype characteristic of MASLD, including significant weight gain, visceral adiposity, glucose intolerance, and pronounced hepatic steatosis, inflammation, and fibrosis. A novel and critical finding of this research is the concurrent development of adverse cardiac remodeling, featuring ventricular hypertrophy and fibrosis. The administration of *L. crispatus* prevented these multi-organ pathologies, highlighting its therapeutic potential through the modulation of key metabolic signaling pathways.

Since MASLD is defined by excessive triglyceride buildup in the liver, this study assessed hepatic lipid accumulation using ultrasound imaging and histopathological analysis. Liver echogenicity, a non-invasive ultrasound-based parameter, serves as an indicator of hepatic steatosis in MASLD patients [39]. In our observations, CDHF-fed mice showed elevated echogenicity at all measured time points. Increased liver echogenicity in MASLD has been identified as a key marker for early diagnosis and management of metabolic disturbances such as dyslipidaemia and insulin resistance [40]. Notably, prior colonization and treatment of *L. crispatus* in CDHF-fed mice led to a consistent reduction in hepatic echogenicity from week 6 to week 12 of the experimental period. These results suggest that *L. crispatus* effectively mitigates hepatic steatosis in CDHF-fed mice, which was further corroborated by histopathological examination. Histopathological analysis revealed pronounced hepatic ballooning, steatosis, and inflammatory cell infiltration in H&E stained liver sections of CDHF fed mice, indicating substantial hepatocellular injury. Additionally, Masson’s trichrome staining confirmed the presence of increased collagen deposition, consistent with progressive hepatic fibrosis. Notably, administration of *L. crispatus* markedly attenuated these pathological alterations, demonstrating recovery across all evaluated parameters, including hepatocellular ballooning, inflammation, steatosis, and fibrosis.

Previous studies have described the association of fatty liver disease with several subclinical cardiac abnormalities, including increased left ventricular (LV) mass, wall thickness, and mass-to-volume ratio in MASLD patients [41]. In the current study, the observed increase in anterior and posterior LV wall thickness up to the 12th week indicates left ventricular hypertrophy. Supporting our findings, VanWagner et al. had reported that MASLD patients developed LV hypertrophy and altered ventricular geometry over a five-year period [42]. Similarly, Cong et al. found reduced LV diastolic diameter in non-obese MASLD patients, a feature typically seen in later stages of ventricular dysfunction [43]. During the early phase of cardiac hypertrophy, reduced end-diastolic volume occurs as thickened cardiac muscles require greater contractile force to pump blood [28]. Enhanced contractility consequently decreases both systolic volume and diameter [44]. Decreased LV diameter and volume during both systole and diastole observed in the CDHF-fed mice suggest the development of cardiac hypertrophy and LV dysfunction. Treatment with *L. crispatus* improved LV wall thickness, diameter, and volume, indicating its protective effect against MASLD-associated cardiac remodeling. Histological analysis also revealed interstitial fibrosis in the hearts of CDHF-fed mice. Excessive fibrosis can contribute to arrhythmias, restrict oxygen delivery by disturbing myocyte perfusion, and reduce ventricular compliance, leading to diastolic dysfunction. Such chronic pathological changes can alter extracellular matrix enzymatic responses and elevate the risk of heart failure [45]. To further evaluate the cardioprotective effects of *L. crispatus*, we measured ejection fraction, fractional shortening, and stroke volume via echocardiography. The ejection fraction represents the percentage of blood expelled from the left ventricle during each heartbeat. CDHF-fed mice demonstrated significant increases in both ejection fraction and fractional shortening. Elevated ejection fraction can arise from mechanisms such as reduced LV end-diastolic volume (LVEDV), increased LV mass-to-volume ratio, and heightened myocardial contractility, and considered as compensatory hyperdynamic state [46, 47]. *L. crispatus*-induced reduction in ejection fraction and fractional shortening is likely a beneficial normalization of this hypercontractility, preventing the progression to heart failure with preserved EF (HFpEF), which is common in metabolic disease. Collectively, echocardiographic results indicate that *L. crispatus* ameliorates both structural and functional cardiac abnormalities in CDHF-fed mice.

To understand the molecular mechanism behind observed benefit of *L. crispatus* in cardiometabolic disease model, we did metabolomics study. By using metabolomics data and structured bioinformatics workflow, we have identified the serum metabolites that altered in diseased state and reversed by *L. crispatus* treatment. Altered metabolites are associated with energy metabolism, oxidative phosphorylation, and lipid signaling. Succinate and fumarate, key intermediates of the tricarboxylic acid (TCA) cycle, were elevated in CDHF group compared to the control group, suggesting a potential metabolic shift towards an altered mitochondrial function and impaired oxidative phosphorylation [48]. Interestingly, *L. crispatus* treatment reversed their levels to normal. Further, the KEGG pathway enrichment analysis revealed significant metabolic alterations, with pathways such as β-fatty acid oxidation and biosynthesis of unsaturated fatty acids, in diseased model and reversed by *L. crispatus* treatment.

Building upon these metabolomic findings, our mechanistic study further explored how disruptions in hepatic β-oxidation contribute to lipid accumulation in the liver. Hepatic β-oxidation is essential for preventing steatosis; however, methionine- and choline-deficient diets suppress β-oxidation and triglyceride clearance. β-oxidation of fatty acids occurs in mitochondria and is regulated by PPARα, PGC-1α, and CPT1α, with SIRT1 serving as a key upstream modulator [49, 50]. SIRT1, an NAD⁺-dependent deacetylase, modulates PGC-1α and is linked with MASLD pathophysiology [51]. Previous research showed that MASLD leads to the downregulation of hepatic SIRT1, PGC-1α, CPT1α, and PPARα expression, and SIRT1 deficiency impairs PPARα signaling and fatty acid oxidation [52–56]. Furthermore, mice lacking SIRT1 showed a significant decrease in the expression of PGC-1 α, impaired the PPAR α signaling and reduction in fatty acid oxidation [57]. Based on our metabolomics data and previous literatures, we focused on the expression of SIRT1, PGC-1α, CPT1α, and PPARα, which are associated with β-oxidation pathway. Our study showed that CDHF feeding disrupted hepatic SIRT1, PGC-1α, CPT1α, and PPARα expression, which was partially restored following *L. crispatus* administration. Recent studies indicate that lipid oxidation pathways in the liver are regulated by AMPK [50]. AMPK enhances SIRT1 activity by elevating cellular NAD⁺ levels and promotes the deacetylation of SIRT1-downstream target such as PGC-1α [58]. Although AMPK can directly phosphorylate PGC-1α [59], phosphorylation followed by deacetylation by SIRT1 is essential for the activation of PGC-1α [60]. Therefore, AMPK activation is recognized as a key mechanism for the therapeutic benefit of *L. crispatus* in cardiometabolic disorder.

In summary, the present study demonstrates that *L. crispatus* not only exhibits antimicrobial activity against clinically relevant pathogens, but also protects against cardiometabolic disorder. Through integrated microbiological, metabolomic and mechanistic analysis, we show that oral supplementation of *L. crispatus* modulates key metabolic signaling pathways to restore hepatic β-oxidation and reduce lipid accumulation. These findings, together with improvements in cardiac metabolic markers, highlight a coordinated gut-liver-heart axis whereby gut microbial interventions influence hepatic metabolism and cardiac health.

## Conclusion

Collectively, this study offers a comprehensive evaluation of *L. crispatus* as a multifunctional probiotic with potent antimicrobial activity and significant benefit to improve cardiometabolic health. Our findings extend its relevance beyond the reproductive tract, highlighting its potential role in promoting cardiometabolic health and preventing MASLD.

## Supplementary Information

Supplementary material 1.

## Data Availability

Complete genome sequences of *L. crispatus* strains S10-5-C2-2, S9-4-C11 and S7-7-C9 are available in the National Centre for Biotechnology Information (https://www.ncbi.nlm.nih.gov/) under accession numbers [GCA\_964656045.1] (https:/www.ebi.ac.uk/ena/browser/view/GCA_964656045.1), [GCA\_964656025.1] (https:/www.ebi.ac.uk/ena/browser/view/GCA_964656025.1), [GCA\_964656035.1] (https:/www.ebi.ac.uk/ena/browser/view/GCA_964656035.1) and Indian Biological Data Centre (https://ibdc.dbtindia.gov.in) accession numbers INGCA000000147, INGCA000000146 and INGCA000000145 respectively.
