# Supplementary material for: Molecular insights into the antimicrobial and cardiometabolic functions of Lactobacillus crispatus isolated from the reproductive tract microbiota of Indian women
Source: J Biomed Sci. 2026 Jan 5;33:7. doi: 10.1186/s12929-025-01207-w (PMC12766959; doi:10.1186/s12929-025-01207-w)
Supplement: Supplementary file 1 — Supplementary material 1. [file 12929_2025_1207_MOESM1_ESM.docx]

**Title: Molecular Insights into the Antimicrobial and Cardiometabolic Functions of *Lactobacillus crispatus* isolated from the Reproductive Tract Microbiota** **of Indian Women**

Shriram Mahajan^1#^, Lekshmi N^2#^, Proxima Dhiman^2^, Manjari Gupta^2^, Pallavi Mudgal^2^, Rajni Yadav^3^, Sudheer Arava^3^, Shinjini Bhatnagar^2^, Nitya Wadhwa^2^, Yashwant Kumar^2^, Daizee Talukdar^2^, Bhabatosh Das^2^*, Sanjay K Banerjee^1^*

^1^Department of Biotechnology, National Institute of Pharmaceutical Education and Research, Guwahati-781101, Assam, India.

^2^ BRIC-Translational Health Science and Technology Institute, Faridabad, Haryana, India

^3^All India Institute of Medical Sciences, New Delhi, India.

**Correspondence Mail ID:** [sanjay@niperguwahati.in](mailto:sanjay@niperguwahati.in), [bhabatosh@thsti.res.in](mailto:bhabatosh@thsti.res.in)

Shriram Mahajan, Email: [shrirammahajan3@gmail.com](mailto:shrirammahajan3@gmail.com), ORCID: 0000-0002-3322-820X

Lekshmi N, Email: [lekshmin@thsti.res.in](mailto:lekshmin@thsti.res.in), ORCID: 0009-0009-8904-3202

Proxima Dhiman, Email: [dhimanproxima@thsti.res.in](mailto:dhimanproxima@thsti.res.in), ORCID: 0009-0002-6576-862X

Manjari Gupta, Email: [manjari@thsti.res.in](mailto:manjari@thsti.res.in), ORCID: [0009-0004-0119-](https://orcid.org/0000-0003-2085-6271?lang=en)5820

Pallavi Mudgal, Email: [pimudgal96@gmail.com](mailto:pimudgal96@gmail.com)

Rajni Yadav, Email: [drrajniyadav@gmail.com](mailto:drrajniyadav@gmail.com)

Sudheer Arava, Email: [aravaaiims@gmail.com](mailto:aravaaiims@gmail.com)

Shinjini Bhatnagar, Email: [shinjini.bhatnagar@gmail.com](mailto:shinjini.bhatnagar@gmail.com)

Nitya Wadhwa, Email: [nitya.wadhwa@thsti.res.in](mailto:nitya.wadhwa@thsti.res.in)

Yashwant Kumar, Email: [y.kumar@thsti.res.in](mailto:y.kumar@thsti.res.in)

Daizee Talukdar, Email: [daizeetalukdar@thsti.res.in](mailto:daizeetalukdar@thsti.res.in) ORCID:[0000-0003-2085-6271](https://orcid.org/0000-0003-2085-6271?lang=en)

Bhabatosh Das, Email: ORCID: [bhabatosh@thsti.res.in](mailto:bhabatosh@thsti.res.in), ORCID:[0009-0001-5501-371X](https://orcid.org/0009-0001-5501-371X)

Sanjay K Banerjee, Email: [sanjay@niperguwahati.in](mailto:sanjay@niperguwahati.in), ORCID: 0000-0002-0008-0480

**# Equal Contribution**

***Corresponding Author’s**

Sanjay K Banerjee

National Institute of Pharmaceutical Education and Research, Guwahati

Department of Biotechnology, Assam, India

Tel: +917042354595

Email: [sanjay@niperguwahati.in](mailto:sanjay@niperguwahati.in)

Bhabatosh Das

BRIC-Translational Health Science and Technology Institute, Faridabad

Department of Biotechnology, Faridabad, India

Tel: +918587010523

Email: [bhabatosh@thsti.res.in](mailto:bhabatosh@thsti.res.in)

**Supplementary Data**

**Supplementary table 1.** List of bacterial and fungal isolates used in the study and their culture conditions

| **Sl no** | **Test strains** | **Strain ID** | **Source** | **Growth media** | **Growth condition** |
| --- | --- | --- | --- | --- | --- |
| 1 | *Lactobacillus crispatus* | S10-5-C2-2 | Human vagina | deMan, Rogosa and Sharpe broth and agar supplemented with 0.001% | 37°C, anaerobic |
| 2 | *Lactobacillus crispatus* | S9-4-C11 | Human vagina | deMan, Rogosa and Sharpe broth and agar supplemented with 0.001% | 37°C, anaerobic |
| 3 | *Lactobacillus crispatus* | S7-7-C9 | Human vagina | deMan, Rogosa and Sharpe broth and agar supplemented with 0.001% | 37°C, anaerobic |
| **Sl no** | **Reference strain** | **Strain ID** | **Source** | **Growth media** | **Growth condition** |
| 1 | *Lacticaseibacillus rhamnosus* | FGL-Lr-GG | BIFILAC GG | deMan, Rogosa and Sharpe broth and agar supplemented with 0.001% | 37°C, anaerobic |
| **Sl no** | **Bacterial pathogens** | **Strain ID** | **Source** | **Growth media** | **Growth condition** |
| 1 | *Acinetobacter baumannii* | B10631 | Blood | Luria-Bertani broth and agar | 37°C, aerobic |
| 2 | *Enterobacter hormaechei* | B10746 | Blood | Luria-Bertani broth and agar | 37°C, aerobic |
| 3 | *Escherichia coli* | B30194 | Blood | Luria-Bertani broth and agar | 37°C, aerobic |
| 4 | *Klebsiella pneumoniae* | B20323 | Blood | Luria-Bertani broth and agar | 37°C, aerobic |
| 5 | *Pseudomonas aeruginosa* | B40129 | Blood | Luria-Bertani broth and agar | 37°C, aerobic |
| 6 | *Salmonella enterica* | VCTN14SAL70 | Feces | Luria-Bertani broth and agar | 37°C, aerobic |
| 7 | *Shigella dysenteriae* | IDH06370 | Feces | Luria-Bertani broth and agar | 37°C, aerobic |
| 8 | *Vibrio cholerae* | N16961 | Feces | Luria-Bertani broth and agar | 37°C, aerobic |
| 9 | *Enterococcus faecalis* | B30539 | Blood | Luria-Bertani broth and agar | 37°C, aerobic |
| 10 | *Staphylococcus aureus* | Abs-B11 | Burn wound | Luria-Bertani broth and agar | 37°C, aerobic |
| 11 | *Staphylococcus haemolyticus* | B20126 | Blood | Luria-Bertani broth and agar | 37°C, aerobic |
| 12 | *Staphylococcus hominis* | B40142 | Blood | Luria-Bertani broth and agar | 37°C, aerobic |
| 13 | *Streptococcus agalactiae* | B10726 | Blood | Luria-Bertani broth and agar | 37°C, aerobic |
| **Sl no** | **Fungal pathogens** | **Strain ID** | **Source** | **Growth media** | **Growth condition** |
| 1 | *Candida albicans* | PI-3 | Feces | Potato dextrose broth and agar | 30°C, aerobic |
| 2 | *Candida glabrata* | B1 | Feces | Potato dextrose broth and agar | 30°C, aerobic |
| 3 | *Candida parapsilosis* | San2 | Feces | Potato dextrose broth and agar | 30°C, aerobic |
| **Sl no** | **Commensals** | **Strain ID** | **Source** | **Growth media** | **Growth condition** |
| 1 | *Lactobacillus gasseri* | S1-F-C2 | Human vagina | deMan, Rogosa and Sharpe broth and agar supplemented with 0.001% | 37°C, anaerobic |
| 2 | *Lactobacillus paragasseri* | S13-C10 | Human vagina | deMan, Rogosa and Sharpe broth and agar supplemented with 0.001% | 37°C, anaerobic |
| 3 | *Lactobacillus jensenii* | S6-12-3 | Human vagina | deMan, Rogosa and Sharpe broth and agar supplemented with 0.001% | 37°C, anaerobic |


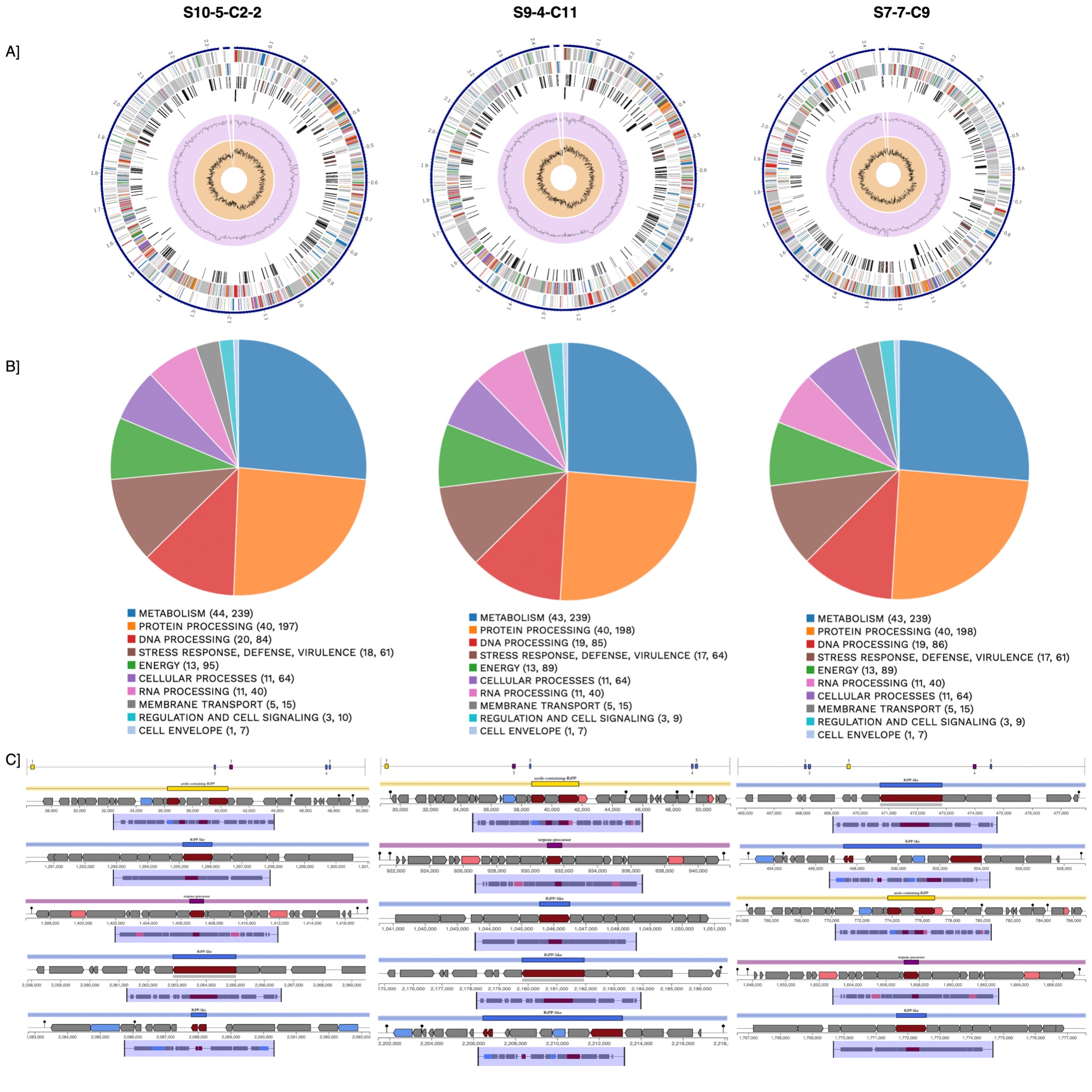


**Supplementary Figure 1. Genome features of the *L. crispatus* isolates. A]** Circular genome maps of the *L. crispatus* isolates S10-5-C2-2, S9-4-C11 and S7-7-C9**.** From outer to inner rings, the contigs, CDS on the forward strand, CDS on the reverse strand, RNA genes, CDS with homology to known stress response, GC content and GC skew. The colors of the CDS on the forward and reverse strand indicate the subsystem that these genes belong to. **B]** COG subsystem classification of proteins encoded by the *L. crispatus* strains **C]** Schematic diagram of the biosynthetic gene clusters (BGCs) identified in *L. crispatus* S10-5-C2-2, S9-4-C11 and S7-7-C9 strains predicted by AntiSMASH.


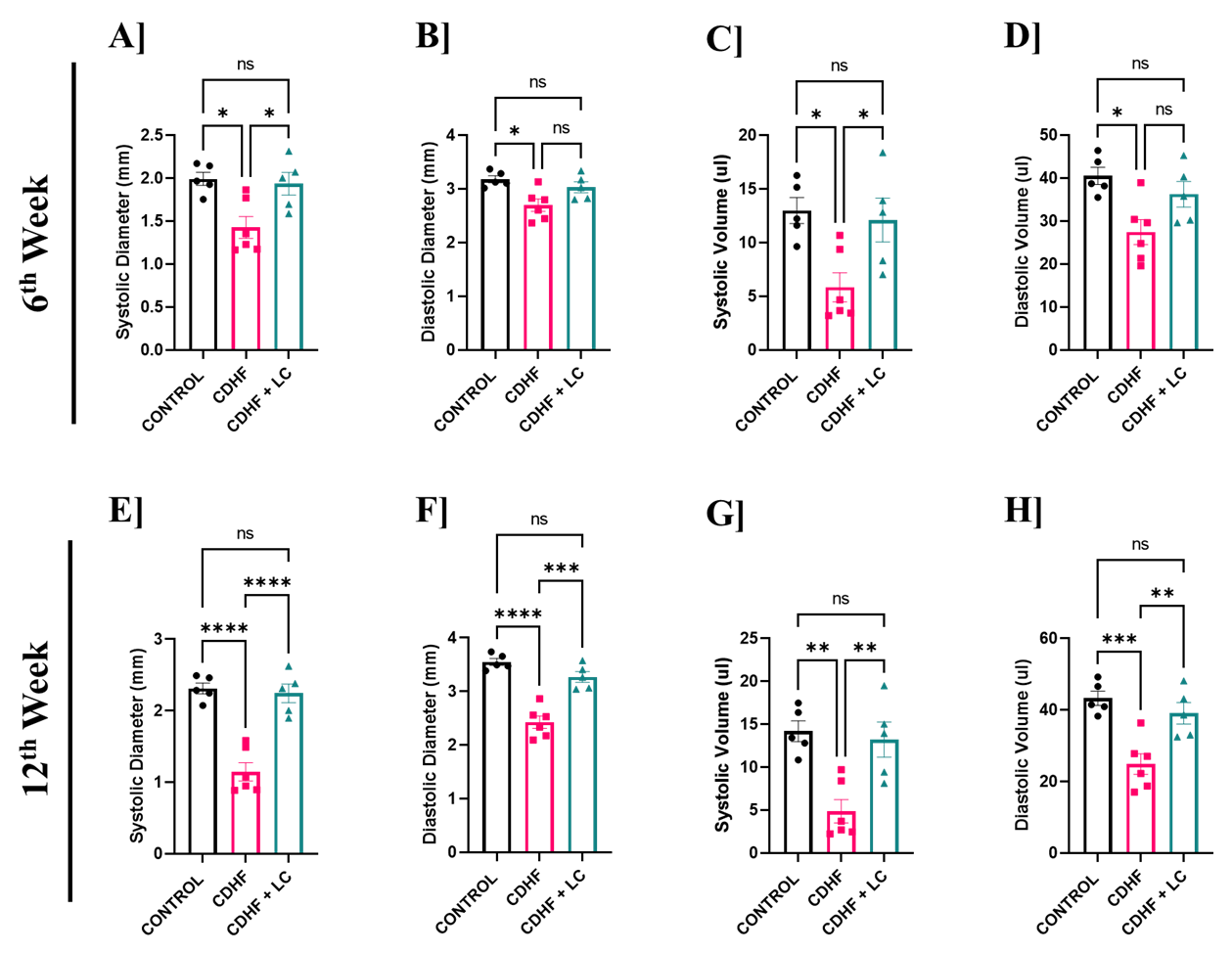


**Supplementary Figure 2.** **Changes in cardiac volumes and diameters measured by echocardiography at 6^th^ and 12^th^ week among Control, CDHF and CDHF+LC.** [A] 6^th^ week left ventricular systolic diameter [B] 6^th^ week left ventricular diastolic diameter [C] 6^th^ week left ventricular systolic volume [D] 6^th^ week left ventricular diastolic volume [E] 12^th^ week s left ventricular systolic diameter [F] 12^th^ week left ventricular diastolic diameter [G] 12^th^ week left ventricular systolic volume [H] 12^th^ week left ventricular diastolic volume. Values are represented as Mean ± SEM. Statistics was done by One-way ANOVA followed by Tukey's test where *p< 0.05, ** p< 0.01, *** p< 0.001, **** p< 0.0001 N=5-6.

**Supplementary table 2.** Key genes involved in different metabolic pathways associated with host-health benefits.

| **KEGG pathway** | **S10-5-C2-2** | | **S7-7-C9** | | **S9-4-C11** | |
| --- | --- | --- | --- | --- | --- | --- |
|  | **Gene ID** | **Gene name** | **Gene ID** | **Gene name** | **Gene ID** | **Gene name** |
| Short chain fatty acid biosynthesis (map00620) | 1_2309 | ppc | 1_682 | ppc | 1_2451 | ppc |
|  | 1_1947 | adhE | 1_327 | adhE | 1_2096 | adhE |
|  | 1_601 | pps | 1_1354 | pps | 1_608 | pps |
|  | 1_2375 | pox1 | 1_749 | pox1 | 1_2520 | pox1 |
|  | 1_1802 | - | 1_179 | - | 1_1947 | - |
|  | 1_1025 | pyk | 1_2083 | pyk | 1_1333 | pyk |
|  | 1_469 | spxB | 1_1218 | spxB | 1_469 | spxB |
|  | 1_1935 | pckA | 1_312 | pckA | 1_2081 | pckA |
|  | 1_907 | fumC | 1_2206 | fumC | 1_1456 | fumC |
|  | 1_100 | accC | 1_857 | accC | 1_101 | accC |
|  | 1_908 | frdA | 1_2205 | frdA | 1_1455 | frdA |
|  | 1_811 | lctO | 1_1615 | lctO | 1_866 | lctO |
|  | 1_809 | ackA | 1_1613 | ackA | 1_864 | ackA |
|  | 1_1653 | ackA | 1_32 | ackA | 1_1799 | ackA |
|  | 1_1781 | atoB | 1_159 | atoB | 1_1927 | atoB |
|  | 1_106 | ldhA | 1_862 | ldhA | 1_106 | ldhA |
|  | 1_1699 | pta | 1_76 | pta | 1_1843 | pta |
|  | 1_323 | ldh | 1_1082 | ldh | 1_332 | ldh |
|  | 1_909 | ldh | 1_2204 | ldh | 1_1454 | ldh |
|  | 1_700 | ldh3 | 1_1453 | ldh3 | 1_707 | ldh3 |
|  | 1_101 | accD | 1_858 | accD | 1_102 | accD |
|  | 1_102 | accA | 1_859 | accA | 1_103 | accA |
|  | 1_684 | acyP | 1_1438 | acyP | 1_692 | acyP |
| Short chain fatty acid biosynthesis (map00640) | 1_100 | accC | 1_857 | accC | 1_101 | accC |
|  | 1_809 | ackA | 1_1613 | ackA | 1_864 | ackA |
|  | 1_1653 | ackA | 1_32 | ackA | 1_1799 | ackA |
|  | 1_1781 | atoB | 1_159 | atoB | 1_1927 | atoB |
|  | 1_1699 | pta | 1_76 | pta | 1_1843 | pta |
|  | 1_323 | ldh | 1_1082 | ldh | 1_332 | ldh |
|  | 1_909 | ldh | 1_2204 | ldh | 1_1454 | ldh |
|  | 1_700 | ldh3 | 1_1453 | ldh3 | 1_707 | ldh3 |
|  | 1_101 | accD | 1_858 | accD | 1_102 | accD |
|  | 1_102 | accA | 1_859 | accA | 1_103 | accA |
| Riboflavin metabolism (map00740) | 1_870 | ribBA | 1_1567 | ribBA | 1_818 | ribBA |
|  | 1_844 | ribBA | - | - | - | - |
|  | 1_872 | ribD | 1_1569 | ribD | 1_820 | ribD |
|  | 1_1367 | ribF | 1_1753 | ribF | 1_1004 | ribF |
|  | 1_1816 | - | 1_193 | - | 1_1961 | - |
|  | 1_85 | - | 1_842 | - | 1_86 | - |
|  | 1_1197 | - | 1_1923 | - | 1_1174 | - |
|  | 1_1927 | nfrA | 1_303 | nfrA | 1_2072 | nfrA |
|  | 1_871 | ribE | 1_1568 | ribE | 1_819 | ribE |
|  | 1_2180 | azr | 1_558 | azr | 1_2327 | azr |
|  | 1_2181 | azo1 | 1_559 | azo1 | 1_2328 | azo1 |
|  | 1_869 | ribH | 1_2225 | ribH | 1_1475 | ribH |
|  | 1_843 | ribH | 1_1566 | ribH | 1_817 | ribH |
| Thiamine metabolism (map00730) | 1_426 | dxs | 1_1176 | dxs | 1_427 | dxs |
|  | 1_1614 | thiI | 1_2354 | thiI | 1_1607 | thiI |
|  | 1_1615 | iscS2 | 1_2355 | iscS2 | 1_1608 | iscS2 |
|  | 1_1572 | iscS | 1_2314 | iscS | 1_1567 | iscS |
|  | 1_1428 | rsgA | 1_1691 | rsgA | 1_942 | rsgA |
|  | 1_2235 | thiD | 1_612 | thiD | 1_2381 | thiD |
|  | 1_1426 | thiN | 1_1693 | thiN | 1_944 | thiN |
|  | 1_368 | adk | 1_1127 | adk | 1_377 | adk |
| Pantothenate biosynthesis (map00770) | 1_469 | spxB | 1_1218 | spxB | 1_469 | spxB |
|  | 1_942 | coaBC | 1_2171 | coaBC | 1_1421 | coaBC |
|  | 1_2029 | coaA | 1_405 | coaA | 1_2174 | coaA |
|  | 1_636 | coaE | 1_1389 | coaE | 1_643 | coaE |
|  | 1_1554 | coaD | 1_2296 | coaD | 1_1549 | coaD |
|  | 1_319 | acpS | 1_1078 | acpS | 1_328 | acpS |
| Biotin metabolism (map00780) | 1_97 | fabF | 1_854 | fabF | 1_98 | fabF |
|  | 1_103 | fabI | 1_860 | fabI | 1_104 | fabI |
|  | 1_96 | fabG | 1_853 | fabG | 1_97 | fabG |
|  | 1_1745 | fabG | 1_122 | fabG | 1_1890 | fabG |
|  | 1_99 | fabZ | 1_856 | fabZ | 1_100 | fabZ |
| Folate biosynthesis (map00790) | 1_1601 | folC | 1_2342 | folC | 1_1595 | folC |
|  | 1_870 | ribBA | 1_1567 | ribBA | 1_818 | ribBA |
|  | 1_844 | ribBA | - | - | - | - |
|  | 1_901 | folA | 1_2212 | folA | 1_1462 | folA |
| Bile acid metabolism (map00120) | 1_825 | cbh | 1_2250 | cbh | 1_1501 | cbh |
| Bile acid metabolism (map00121) | 1_825 | cbh | 1_2250 | cbh | 1_1501 | cbh |
| Vitamin B6 biosynthesis (map00750) | 1_1243 | thrC | 1_1879 | thrC | 1_1130 | thrC |
